# Targeting *Meloidogyne incognita* with *Clonostachys rosea*: Strain-Dependent Biocontrol Performance and Yield Effects Under Greenhouse Conditions

**DOI:** 10.3390/plants15182809

**Published:** 2026-09-13

**Authors:** Tobias Stucky, Lara de Gianni, Jakob Egger, Jürgen Kraus, Paul Dahlin, Andrea C. Ruthes

**Affiliations:** 1 Entomology and Nematology, Plant Protection, Agroscope, Müller-Thurgau-Strasse 29, 8820 Wädenswil, Switzerland; 2 Vegetable-Production Extension, Plants and Plant Products, Agroscope, Müller-Thurgau-Strasse 29, 8820 Wädenswil, Switzerland; 3 Mycology, Plant Protection, Agroscope, Route de Duillier 60, 1260 Nyon, Switzerland

**Keywords:** *Clonostachys rosea*, root-knot nematode, *Meloidogyne incognita*, biological control, isolate-specific effects, nematostatic activity, soil-borne pathogens, tomato

## Abstract

The root-knot nematode *Meloidogyne incognita* poses a major challenge to global crop production, yet sustainable management options remain limited. We evaluated a collection of *Clonostachys rosea* isolates for their antagonistic potential using a stepwise approach that included in vitro assays, in planta tests, and greenhouse trials. Clear strain-dependent differences in biocontrol activity were observed across the different experimental stages. In vitro, several isolates strongly inhibited egg development and suppressed second-stage juvenile (J2) motility, with the magnitude of inhibition varying among isolates, concentrations, and incubation times. Changes in motility at some intermediate concentrations indicated that inhibition was not uniformly sustained over time. In cucumber assays, several isolates reduced root galling, with stronger suppression generally observed following J2 than egg inoculation. Selected isolates were subsequently evaluated under greenhouse conditions on tomato, where 1308, 1910, and 2084 produced reductions in root galling at selected early and mid-season assessments. Although differences in galling diminished under prolonged nematode pressure, selected isolates produced harvest-specific increases in individual fruit weight, whereas cumulative fruit number and fruit weight did not differ significantly among treatments. These results indicate that effective *C. rosea* isolates can contribute to integrated nematode management by reducing early nematode infection and potentially supporting plant performance, even when long-term nematode suppression is limited. Our findings emphasize the importance of multi-stage screening and targeted isolate selection to identify fungal antagonists with activity across different experimental conditions.

## 1. Introduction

The use of *Clonostachys* species, particularly *Clonostachys rosea*, as biological control agents against plant-parasitic nematodes has gained increasing attention amid the global shift toward more sustainable agricultural practices [1,2,3,4]. Plant-parasitic nematodes, including the root-knot nematode *Meloidogyne incognita*, are responsible for substantial crop losses worldwide, contributing to more than 30% of yield reductions in affected systems [5]. Controlling these pests remains challenging, particularly as chemical nematicides face growing restrictions due to environmental and health concerns [6,7], which has intensified the search for effective bio-based alternatives compatible with integrated pest management strategies.

In Switzerland, the only registered bio-based nematicide is *Purpureocillium lilacinum* strain 251 [8], a filamentous fungus that parasitizes nematode eggs and juveniles [9]. This highlights the limited availability of biocontrol solutions for nematode management. *M. incognita* is one of the most destructive agricultural pests globally, with an exceptionally broad host range including tomato, eggplant, cucumber, melon, carrot, lettuce, pepper, soybean and potato [10]. Its life cycle completes in approximately four weeks under favorable conditions, progressing through four juvenile stages (J1–J4) before reaching adulthood. Eggs, laid in a gelatinous matrix primarily outside the root cortex, give rise to the first-stage juvenile (J1), which molts into the infective second-stage juvenile (J2). These motile J2 hatch and actively search for host roots, penetrate near the root tip, migrate intercellularly to the vascular cylinder, and induce multinucleated giant cells as specialized feeding sites. Sedentary juveniles develop through the third and fourth stages, characterized by increased swelling, while adult females remain pear-shaped and sedentary; males revert to a vermiform, mobile form. Reproduction is predominantly mitotic parthenogenesis, with males contributing rarely [10].

Among candidate biocontrol agents, *C. rosea* has demonstrated strong antagonistic activity against several economically important nematodes, including *M. incognita*, *Pratylenchus* spp., *Heterodera* spp., as well as other soil-borne pathogens [2,11,12,13,14]. The antagonistic activity of nematophagous fungi can involve several mechanisms, including direct parasitism, antibiosis, and the production of extracellular hydrolytic enzymes and other bioactive compounds [15,16]. For *C. rosea*, proteases and other hydrolytic enzymes, including chitinases and glucanases, have been reported [12,17,18], while fungal secondary metabolites may also contribute to nematode inhibition [15,19,20]. Importantly, these mechanisms are not necessarily expressed to the same extent among isolates, and differences in fungal traits may contribute to variation in biocontrol efficacy [18,21].

Several bioactive compounds, including polyketides, peptaibols, and cyclosporins, have been identified in different *C. rosea* isolates [11,18,19,20,21,22]. Nematotoxic compounds produced by *C. rosea* have also recently been investigated specifically in relation to *M. incognita*, further supporting the potential role of fungal metabolites in nematode suppression [20]. These metabolites likely contribute to nematode suppression, either by directly affecting motile juveniles and eggs or by modulating soil microbial interactions. However, their role in isolate-specific antagonism under variable soil conditions remains largely unexplored. In addition, the biological activity of secreted compounds may depend on their concentration and persistence, which may partly explain differences between activity observed under controlled in vitro conditions and that observed in soil. Understanding these differences is critical, as bioactive compound production and activity can vary widely among isolates, influencing their effectiveness in field conditions.

Despite the documented potential of *C. rosea*, most studies have focused on a limited number of isolates, including commercial isolates, with relatively little attention given to the diversity of isolates available from different geographical and ecological origins [2,14]. Variations in enzyme secretion, metabolite production, and environmental tolerance can substantially affect biocontrol performance [13,14]. Recent studies have further demonstrated substantial natural variation among *C. rosea* isolates in their antagonistic activity against plant-parasitic nematodes, supporting the importance of isolate selection rather than assuming uniform biocontrol performance across the species [21]. Therefore, evaluating isolates from diverse origins and ecological backgrounds may help identify isolates with distinct and potentially valuable biocontrol traits for use in agricultural systems.

The nematode developmental stage may also influence the outcome of fungal antagonism. Eggs are protected by their eggshell and surrounding matrix, whereas J2 are motile and directly exposed to their surrounding environment. An isolate that is effective against J2 may therefore not necessarily show the same level of activity against eggs. Evaluating both developmental stages can provide a broader assessment of isolate-specific biocontrol potential and may help distinguish effects on nematode development from effects on motility or infection.

In this study, we evaluated a diverse collection of *C. rosea* isolates from the Agroscope Mycoscope database for their potential to suppress *M. incognita* across in vitro, in planta, and greenhouse conditions. We aimed to compare isolate performance across nematode developmental stages and experimental conditions and to identify isolates with promising activity for further development as biological control agents against *M. incognita*.

## 2. Results

### 2.1. In Planta Screening of Clonostachys rosea Isolates Against Meloidogyne incognita

In planta assays revealed clear isolate-dependent differences in the ability of *C. rosea* isolates to suppress *M. incognita* infection, with the effect of inoculum type varying among treatments. Overall, root galling was lower following J2 inoculation than following egg inoculation (Wilcoxon rank-sum test: W = 10,181, *p* = 7.19 × 10^−15^). An ordinal logistic regression further revealed a significant Treatment × Inoculum interaction (likelihood-ratio test: χ^2^ = 102.73, *df* = 22, *p* < 0.001), indicating that the effect of inoculum type on galling differed among treatments. Model-based contrasts showed significantly lower GI following J2 than egg inoculation for 12 of the 23 treatments after Holm correction (*p*adj = 3.33 × 10^−11^–0.0157), including isolates 1952, 1308, 1923, 560, 2724, 1910, 2189, 2676, 2084, 2132, 2135, and 2725. No significant Egg–J2 differences were detected for the remaining treatments. PHP1701, the positive control, showed numerically lower galling than the nematode control under both inoculum conditions, although these differences were not statistically significant.

**Figure 1:**
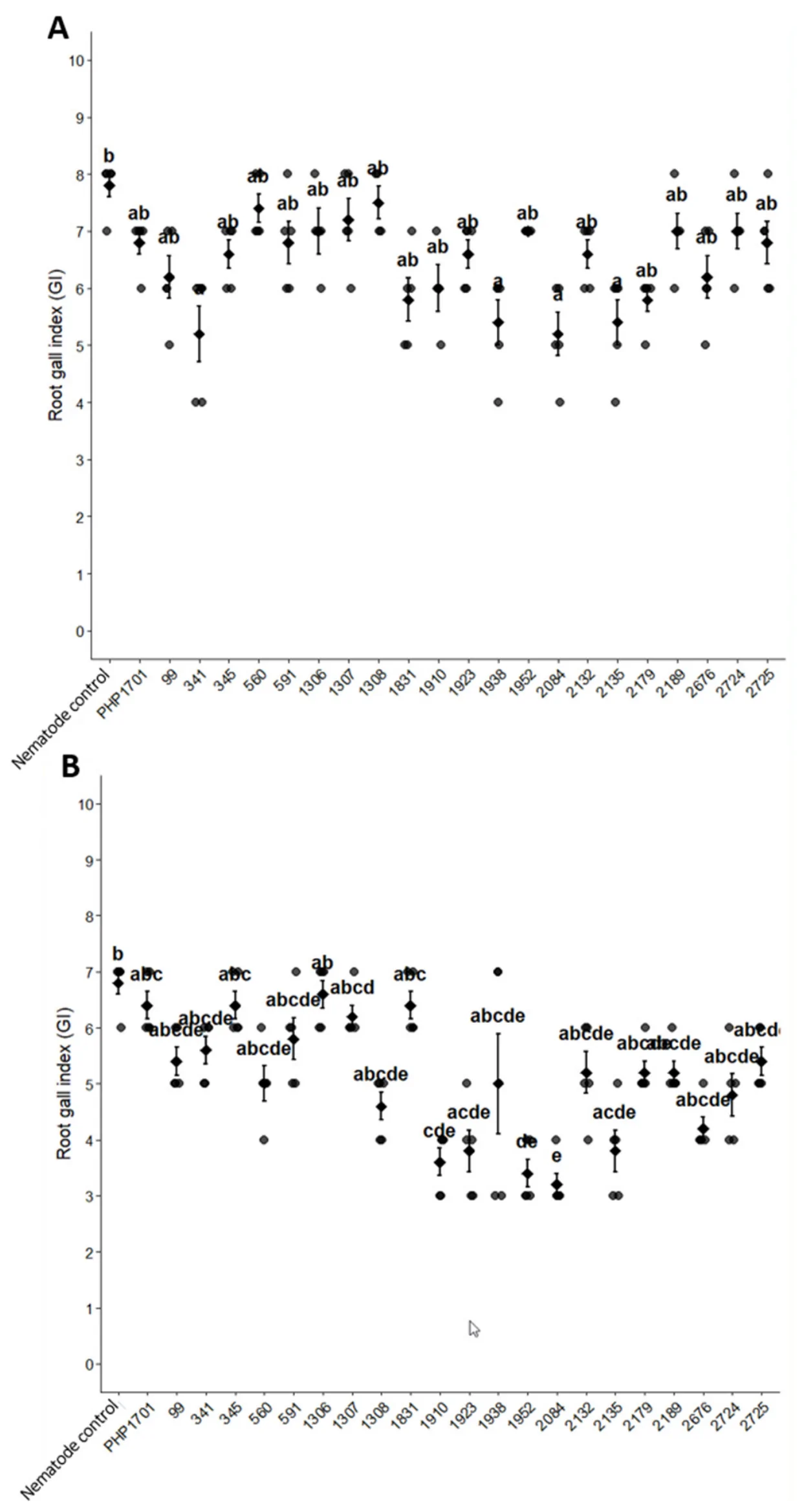
Gall index (GI) of cucumber seedlings after treatment with *Clonostachys rosea* isolates, 35 days (egg inoculation, (**A**)) or 28 days (J2 inoculation, (**B**)) after infection with *M. incognita* (*n* = 5). Negative control lacked fungal treatment, while PHP1701 (*C. rosea*) served as the positive control. Individual points represent individual plants, diamonds indicate treatment means, and vertical error bars represent ±SE. Different letters indicate significant differences according to Dunn’s post-hoc test with Holm adjustment (α = 0.05).

When cucumber seedlings were inoculated with eggs, root galling differed significantly among treatments (Kruskal–Wallis test: χ^2^ = 64.22, *df* = 22, *p* < 0.001). The nematode control had a mean GI of 7.8 ± 0.20, while isolates 2084, 341, 1938, and 2135 showed significantly lower galling, with mean GI values of 5.2 ± 0.37, 5.2 ± 0.49, 5.4 ± 0.40, and 5.4 ± 0.40, respectively (Dunn–Holm, *p*adj = 0.0089–0.0251). No significant differences from the nematode control were detected for the remaining treatments (Figure 1A; Supplementary Table S2). Following J2 inoculation, root galling also differed significantly among treatments (Kruskal–Wallis test: χ^2^ = 83.53, *df* = 22, *p* < 0.001). The nematode control had a mean GI of 6.8 ± 0.20, whereas isolates 1910, 1923, 1952, 2084, and 2135 significantly reduced galling relative to the nematode control (Dunn–Holm, *p*adj = 0.0025–0.0322). The lowest gall index was recorded for isolate 2084 (GI = 3.2 ± 0.20), followed by isolates 1952 (3.4 ± 0.25) and 1910 (3.6 ± 0.25). No significant difference from the nematode control was detected for the remaining treatments (Figure 1B; Supplementary Table S2).

Shoot and root biomass responses varied depending on the inoculum type. In egg-inoculated plants, shoot fresh weight differed significantly among treatments (one-way ANOVA, *F*_22,89_ = 1.87, *p* = 0.0213); however, no significant differences were detected between individual treatments following Tukey’s HSD adjustment (all adjusted *p* > 0.05). Root fresh weight was not significantly affected by treatment (*F*_22,89_ = 1.58, *p* = 0.0705). Similarly, in J2-inoculated plants, shoot fresh weight showed a significant overall treatment effect (*F*_22,85_ = 1.94, *p* = 0.0166), but no significant pairwise differences were detected after Tukey adjustment. Root fresh weight did not differ significantly among treatments (*F*_22,84_ = 1.19, *p* = 0.276) (Supplementary Figure S2; Supplementary Table S4). Direct comparisons between inoculation types revealed significant differences in shoot fresh weight for six treatments: 1306, 1831, 1910, 2135, 2179, and 345 (Holm-adjusted *p* < 0.05; Supplementary Table S3). Shoot fresh weight was higher following J2 inoculation for isolates 1306, 1910, 2135, 2179, and 345, whereas isolate 1831 showed higher shoot fresh weight following egg inoculation. No significant differences between inoculation types were detected for the remaining treatments. Although the combined analysis revealed a significant Treatment × Inoculum interaction for root fresh weight (*F*_22,174_ = 1.79, *p* = 0.0208), post-hoc Egg-vs.-J2 comparisons with Holm correction showed that this difference was attributable primarily to the nematode control, while none of the *C. rosea* treatments differed significantly between inoculation types.

Based on their overall galling-suppression profiles across the two inoculation methods, isolates 1308, 1910, 1923, 1952, 2084, 2135, and 2676 were selected for further in vitro metabolite assays.

### 2.2. In Vitro Culture Filtrate Assays

**Figure 2:**
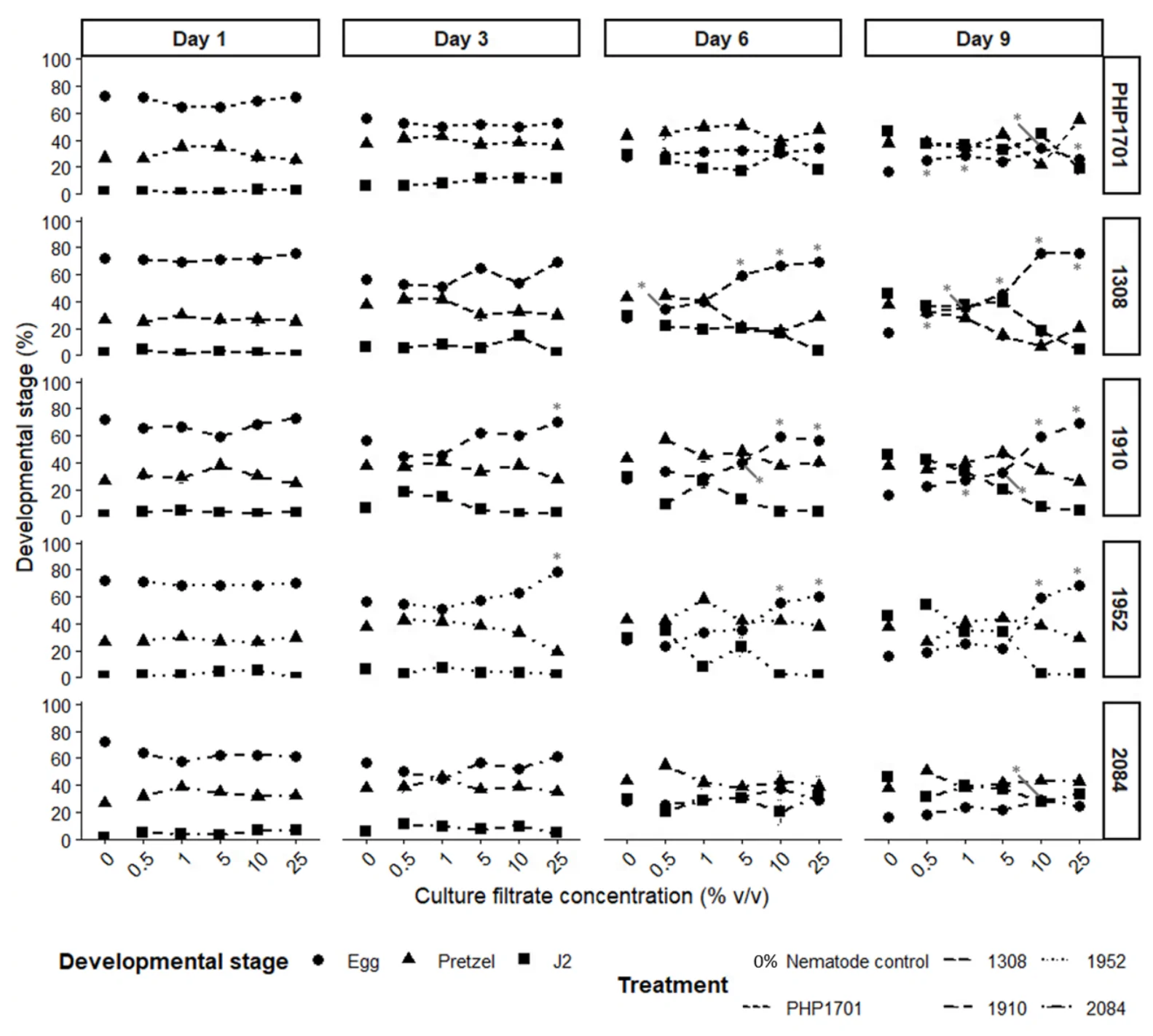
Antibiosis assay showing the effects of increasing concentrations (0, 0.5, 1.0, 5.0, 10 or 25% *v*/*v*) of *Clonostachys rosea* culture filtrates from isolates PHP1701, 1308, 1910, 1952, and 2084 on *Meloidogyne incognita* egg development after 1, 3, 6, and 9 days of incubation. The nematode control is shown alongside each isolate for comparison. Data are presented as mean percentages ± SE of eggs (•), pretzels (▴), and second-stage juveniles (J2; ▪) across biological replicates. Statistical comparisons of egg development between each isolate–concentration treatment and the nematode control were performed within each day using a binomial generalized linear model, with Holm adjustment for multiple comparisons. Adjusted *p* < 0.05 was considered statistically significant (*).

Culture filtrates from the selected isolates were evaluated for their effects on *M. incognita* egg development and J2 motility. In the negative control, eggs progressed through the classical egg → pretzel → J2 sequence, reaching approximately 46% J2 by day 9, establishing a baseline for comparison (Figure 2; Supplementary Table S5).

The effects of culture filtrates on egg development varied among isolates, concentrations, and observation times (Figure 2; Supplementary Table S5). Overall, the inhibitory effects became more pronounced at later observation times and, for several isolates, at higher filtrate concentrations. Isolate 1308 showed the strongest suppression of egg development, particularly at 10% and 25%, with a high proportion of individuals remaining in the egg stage and J2 emergence limited to approximately 2–4% by day 9. Significant differences from the nematode control were observed at these concentrations.

Isolates 1910 and 1952 also markedly delayed egg development at higher concentrations, resulting in significantly reduced progression through the developmental stages compared with the nematode control. By day 9, the effects were particularly pronounced at 10% and 25% filtrate concentrations. Isolate 1923 also affected egg development, although its effects were generally less pronounced. In contrast, isolates 2084, 2135, 2676, and PHP1701 showed weaker or more variable responses across concentrations and observation times. For PHP1701, J2 emergence reached 19.3% by day 9, compared with 46.2% in the untreated nematode control.

Overall, the results demonstrated isolate- and concentration-dependent differences in the effects of culture filtrates on *M. incognita* egg development, with the strongest suppression observed for isolates 1308, 1910, and 1952 at the higher concentrations (Figure 2; Supplementary Table S5).

**Figure 3:**
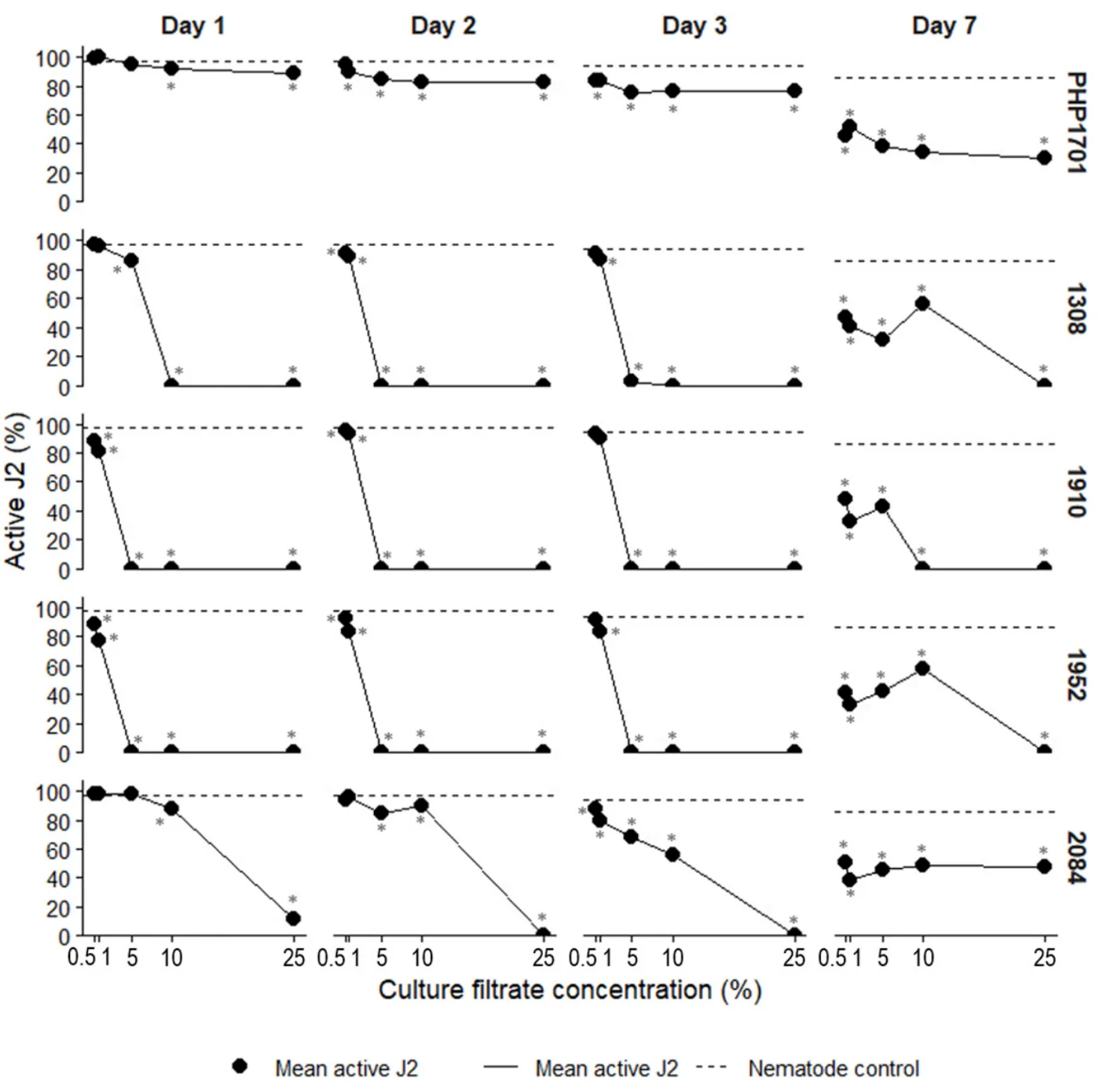
Antibiosis assay showing the effect of different concentrations (0.5, 1.0, 5.0, 10 or 25% media, *v*/*v*) of *Clonostachys rosea* culture filtrates from isolates PHP1701, 1308, 1910, 1952, and 2084 on *Meloidogyne incognita* J2 motility after 1, 2, 3, and 7 days of incubation. Points represent mean percentages of active J2, and error bars represent the standard error (SE) of replicates (*n* = 4). Connecting lines are included as visual guides between concentrations. The dashed horizontal line represents the corresponding nematode control. Statistical comparisons between each filtrate treatment and the nematode control were performed separately for each day using bias-reduced binomial logistic regression, with Holm adjustment for multiple comparisons. Adjusted *p* < 0.05 was considered statistically significant (*).

J2 motility assays showed a progressive reduction in motility following exposure to *C. rosea* culture filtrates, with the magnitude of inhibition varying among isolates, concentrations, and incubation times. Nematode control J2 remained highly active throughout the experiment, with mean activity declining from 97.4% on day 1 to 86.2% on day 7 (Supplementary Table S6). In contrast, several culture filtrates significantly reduced J2 activity compared with the nematode control, particularly at concentrations of ≥5%. Isolate 1308 showed a pronounced effect, with significant reductions in active J2 already observed at 0.5% on day 2 and at 1% from day 3 onward, while concentrations of 5–25% resulted in strong or complete inhibition of motility (Figure 3; Supplementary Table S6). Isolates 1910, 1952, and 2084 likewise caused strong inhibition at higher concentrations, although the onset and magnitude of the response differed among isolates and incubation times (Figure 3; Supplementary Table S6). Isolates 1923, 2135, and 2676 also reduced J2 activity, with the strongest effects generally observed at higher concentrations and after longer exposure periods (Supplementary Figure S4; Supplementary Table S6). By day 7, all tested filtrate treatments showed significantly lower J2 activity than the nematode control after Holm adjustment. At some intermediate concentrations, partial recovery of apparent motility was observed, indicating that the response was not uniformly monotonic across concentrations.

**Figure 4:**
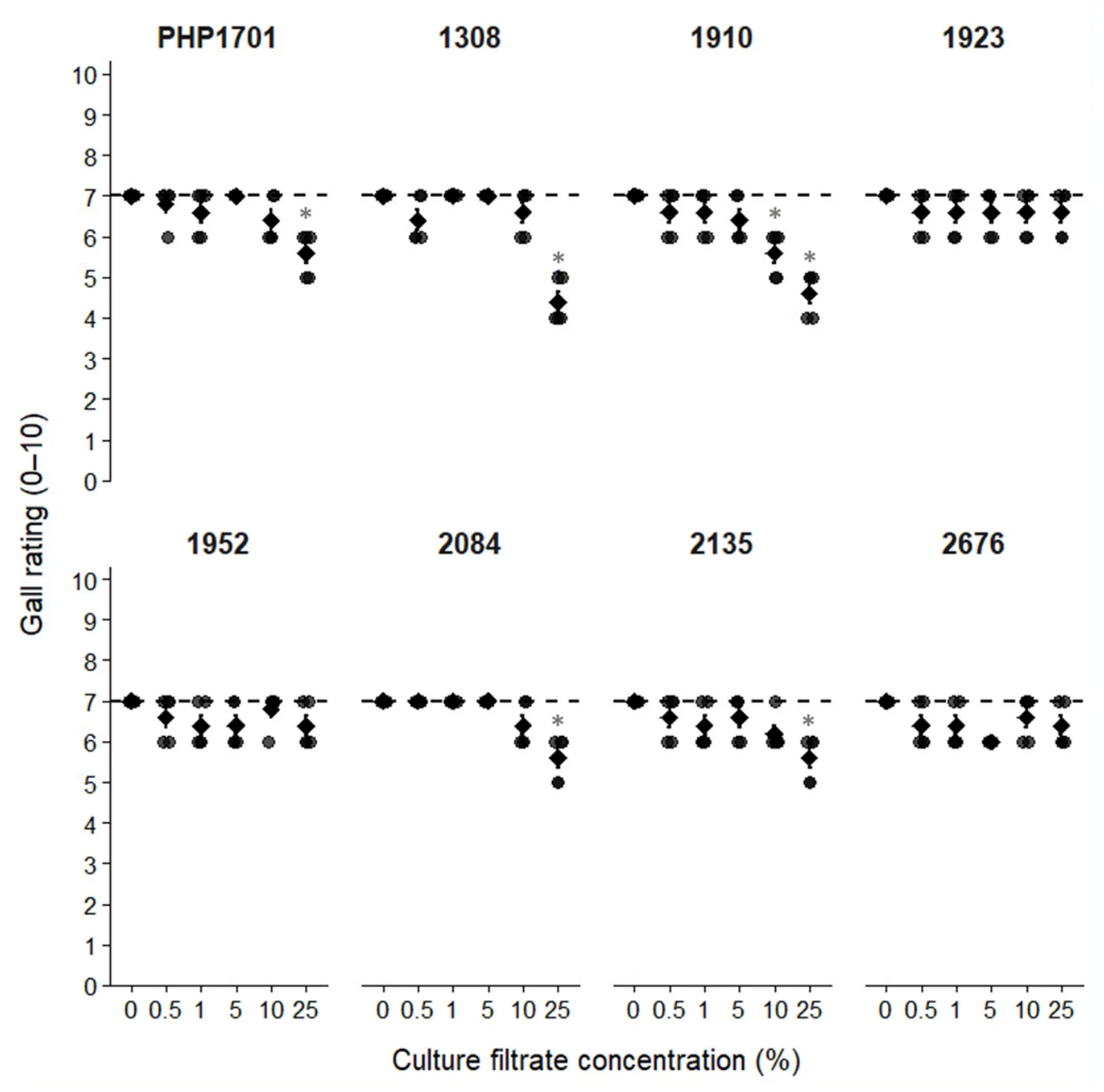
Gall index (GI) of cucumber seedlings after *Meloidogyne incognita* J2 were incubated for 7 days on different *Clonostachys rosea* isolates’ culture filtrates at different concentrations (0.5, 1.0, 5.0, 10 or 25% media, *v*/*v*). After 21 days of J2 inoculum, GI was recorded (*n* = 3). Negative control (0 followed by dashed line) lacked fungal treatment, while PHP1701 (*C. rosea*) served as the positive fungal control. Points (•) represent individual replicates, ⬩ represents the mean, and error bars indicate standard errors. Gall index was analyzed using the Kruskal–Wallis test. Asterisks indicate significant differences from the nematode control according to Dunn’s post-hoc test with Holm adjustment (* *p* < 0.05).

When J2 pre-exposed to culture filtrates for 7 days were subsequently inoculated onto cucumber seedlings, gall ratings differed significantly among treatments (Kruskal–Wallis test: χ^2^ = 135.11, *df* = 48, *p* < 0.001). Dunn’s post-hoc tests with Holm correction revealed significant reductions in gall ratings compared with the nematode control for isolates 1308 and 1910 at 25% concentration, isolate 1910 at 10%, and isolates 2084, 2135, and PHP1701 at 25% concentration (adjusted *p* ≤ 0.0308 Figure 4; Supplementary Table S7). The strongest reductions were observed for isolates 1308 and 1910 at 25%, with mean gall ratings of 4.40 ± 0.25 and 4.60 ± 0.25, respectively, compared with 7.00 ± 0.00 in the nematode control.

Shoot fresh weight was significantly affected by treatment (*F*_8164_ = 8.29, *p* < 0.001), whereas the main effect of culture filtrate concentration was not significant (*F*_4164_ = 0.86, *p* = 0.491). However, a significant treatment × concentration interaction was detected (*F*_28_,_164_ = 2.48, *p* < 0.001), indicating that shoot fresh weight responses to culture filtrate concentration varied among isolates. Tukey’s post-hoc comparisons identified significant differences among specific treatment × concentration combinations (Supplementary Table S7). Root fresh weight was also significantly affected by treatment (*F*_8196_ = 5.92, *p* < 0.001), with significant differences among treatment means identified by Tukey’s post-hoc test (Supplementary Table S7).

Considering the overall responses observed in the culture-filtrate assays, including effects on gall formation and plant biomass, isolates 1308, 1910, 1952, and 2084 were selected for further evaluation under greenhouse conditions.

### 2.3. Greenhouse Evaluation of Selected Clonostachys rosea Isolates

**Figure 5:**
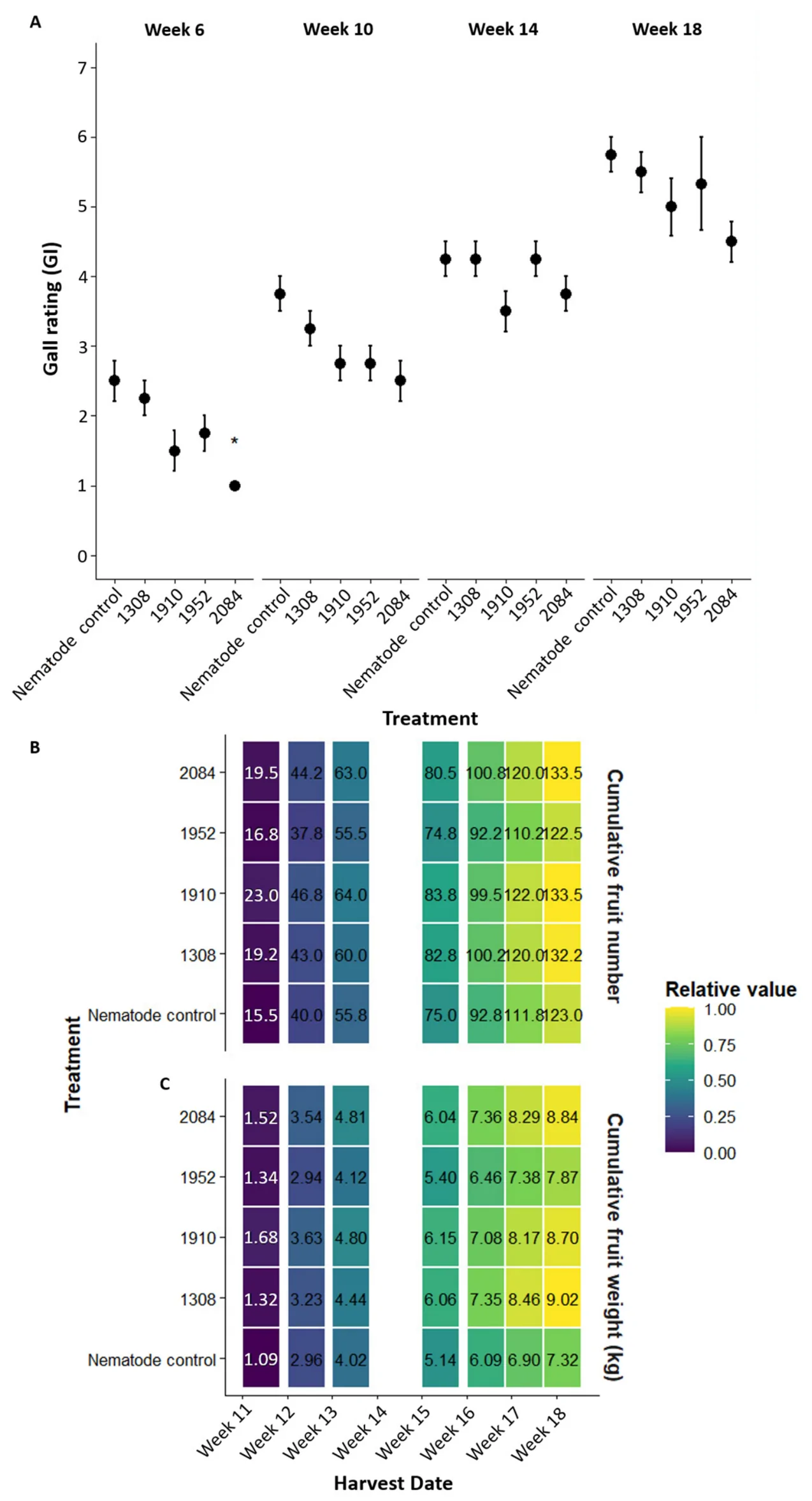
Large-greenhouse trial: (**A**) root gall rating and heatmap of cumulative tomato fruit production in terms of fruit number (**B**), and fruit weight (**C**) across treatments. Root gall rating values represent the mean ± SD per treatment. The heatmap shows cumulative fruit number per plant and cumulative fruit weight per plant (kg) across successive harvest dates. Values in the heatmap represent treatment means calculated from four plants per treatment. Numbers within cells indicate the mean cumulative value, while color intensity represents the relative magnitude of each metric. The heatmap provides a descriptive visualization of the temporal development of cumulative fruit production; statistical comparisons among treatments are presented separately in the corresponding yield analysis Supplementary Table S12. Adjusted *p* < 0.05 was considered statistically significant (*).

In the small-scale greenhouse trial, none of the selected *C. rosea* isolates significantly reduced root galling compared with the untreated control (Kruskal–Wallis test, χ^2^ = 6.16, *df* = 4, *p* = 0.188; Supplementary Figure S5A; Supplementary Table S8) or nematode reproduction compared with the untreated control (Supplementary Figure S5; Supplementary Table S8). Mean root gall indices (GI) ranged from 4.75 to 5.75 across treatments and remained comparable to the untreated control. Root fresh weight differed significantly among treatments overall (one-way ANOVA, *F*_4,35_ = 2.76, *p* = 0.043), with mean values ranging from 6.0 to 7.2 g; however, none of the isolates differed significantly from the nematode control in Tukey-adjusted pairwise comparisons (*p* ≥ 0.264; Supplementary Figure S5B; Supplementary Table S8). Nematode reproduction was not significantly affected by any of the isolates. Mean egg mass numbers ranged from 461 to 602 per plant (one-way ANOVA, *F*_4,15_ = 0.69, *p* = 0.608), while total egg counts ranged from approximately 293,000 to 377,000 eggs per plant (one-way ANOVA, *F*_4,15_ = 0.69, *p* = 0.608). Similarly, the number of eggs per egg mass did not differ significantly among treatments, with mean values ranging from 594 to 757 eggs per egg mass (one-way ANOVA, *F*_4,15_ = 1.15, *p* = 0.372; Supplementary Figure S5C–E; Supplementary Table S8). In the large-scale greenhouse trial, treatment effects on root galling were significant at weeks 6 and 10 but not at weeks 14 and 18 (Kruskal–Wallis tests: χ^2^ = 12.10, *p* = 0.0166; χ^2^ = 9.50, *p* = 0.0497; χ^2^ = 6.33, *p* = 0.176; and χ^2^ = 6.11, *p* = 0.191, respectively). Dunn’s post-hoc comparisons with the nematode control, adjusted using the Holm method, showed that isolate 2084 significantly reduced galling at week 6 (*p*adj = 0.0082) and week 10 (*p*adj = 0.0258). No other isolate differed significantly from the nematode control at any assessment date (Figure 5A; Supplementary Table S9).

Vegetative growth, assessed by branch length, increased significantly over time (Date: *F*_3,60_ = 1287.03, *p* < 0.001) but was not affected by fungal treatment (Treatment: *F*_4,60_ = 0.69, *p* = 0.601). The Treatment × Date interaction was also not significant (*F*_12,60_ = 1.11, *p* = 0.371), indicating that treatments did not alter the trajectory of branch growth over the experimental period (Supplementary Table S10).

Soil nematode population densities increased throughout the experiment across all treatments. No significant differences in juvenile densities among treatments were detected at any sampling date (Kruskal–Wallis tests, *p* > 0.05). At week 18, soils treated with isolate 2084 showed the lowest mean juvenile density (1404 ± 225.9 J2 per 100 cm^3^ soil), compared with 2772 ± 338.2 J2 per 100 cm^3^ soil in the nematode control; however, this difference was not statistically significant (*p* = 0.134). Isolates 1308, 1910, and 1952 likewise showed numerically lower juvenile densities than the control, but these differences were not significant (Supplementary Table S11).

Despite the convergence of gall index later in the season, fungal treatments showed some harvest-specific effects on tomato fruit production. Cumulative fruit number and cumulative fruit weight did not differ significantly among treatments (*F*_4,15_ = 1.07, *p* = 0.406 and *F*_4,15_ = 2.10, *p* = 0.131, respectively), although cumulative fruit weight was numerically higher than in the nematode control by 23.2%, 18.9%, and 20.8% for isolates 1308, 1910, and 2084, respectively (Figure 5B; Supplementary Table S12). Fruit number per harvest was not significantly affected by treatment (χ^2^_4_ = 6.84, *p* = 0.145) but varied among harvest stages (χ^2^_6_ = 21.71, *p* = 0.0014), with no treatment × harvest-stage interaction (χ^2^_24_ = 13.70, *p* = 0.953). In contrast, individual fruit weight was significantly affected by treatment (χ^2^_4_ = 12.36, *p* = 0.0148) and harvest stage (χ^2^_6_ = 177.37, *p* < 0.001), with a significant treatment × harvest-stage interaction (χ^2^_24_ = 50.55, *p* = 0.0012). Compared with the nematode control, isolate 1952 produced heavier fruits at week 6, isolates 1308 and 2084 produced heavier fruits at weeks 14 and 15, isolate 1308 produced heavier fruits at week 16, and isolate 1910 produced heavier fruits at week 17 (Supplementary Table S13).

## 3. Discussion

Our study demonstrates that the biocontrol activity of *Clonostachys rosea* against *Meloidogyne incognita* is highly isolate-dependent and varies according to the nematode developmental stage, application method, and experimental system. Although several isolates showed strong antagonistic activity during in vitro and early in planta screening, these effects became less pronounced under greenhouse conditions, emphasizing the complexity of translating laboratory efficacy into sustained biological control in soil.

### 3.1. Differences in Suppression Between Egg- and J2-Inoculated Plants

Greater reductions in root galling were observed following J2 inoculation than following egg inoculation. However, this difference should be interpreted cautiously because the two inoculum systems were not directly equivalent: plants received 250 J2 or 300 eggs and were evaluated after 28 or 35 days respectively. The different durations were selected based on the developmental dynamics of *M. incognita*. In a preliminary assessment, 78.8% ± 2.12% of the initially inoculated eggs had developed into J2 after 14 days (Supplementary Table S14), supporting the use of a longer period for egg-inoculated plants to allow sufficient development and infection by the resulting J2. The significant Treatment × Inoculum interaction further indicates that the magnitude of the inoculum-dependent response varied among *C. rosea* isolates. Thus, the greater suppression observed following J2 inoculation may reflect the combined effects of nematode developmental stage, inoculum quantity, fungal exposure, and experimental duration, and a strictly stage-specific effect cannot be established from the present experimental design.

Nevertheless, the observed pattern is consistent with previous findings indicating that nematode eggs are relatively protected by their chitinous shell and gelatinous matrix, whereas motile J2 are more exposed to fungal hyphae and potentially to lytic enzymes, and secondary metabolites produced by the fungus [11,20]. The variation among isolates further suggests that the efficacy of *C. rosea* against *M. incognita* is strongly influenced by isolate-specific characteristics. Isolates 2084 and 2135 showed the most consistent suppression across the two inoculation approaches, whereas other isolates showed stronger effects under only one inoculation condition. In particular, isolate 2084 consistently reduced galling under both inoculation conditions and produced the lowest gall index following J2 inoculation, making it a promising candidate for further investigation. However, confirmation under additional experimental conditions will be necessary to establish the robustness of this effect.

The biomass responses provided limited evidence for consistent isolate-specific effects on plant growth within either inoculation system. Although shoot fresh weight showed significant overall treatment effects under both egg and J2 inoculation, these effects were not resolved into significant differences between individual treatments after multiplicity-adjusted Tukey comparisons. Root fresh weight was unaffected by treatment under either inoculation condition. Direct comparison between inoculation types nevertheless revealed significant differences in shoot fresh weight for six isolates (1306, 1831, 1910, 2135, 2179, and 345), indicating that the shoot biomass response differed between inoculation systems for these treatments. Shoot fresh weight was higher following J2 inoculation for isolates 1306, 1910, 2135, 2179, and 345, whereas isolate 1831 showed higher shoot fresh weight following egg inoculation. These differences should nevertheless be interpreted cautiously because the two inoculation systems differed in inoculum type, inoculum quantity, and assessment time. Importantly, the Egg-vs.-J2 differences do not indicate that these isolates significantly increased or reduced shoot biomass relative to the nematode control, as no significant pairwise treatment differences were detected within either inoculation experiment.

A significant Treatment × Inoculum interaction was also detected for root fresh weight, indicating that the effect of inoculation type was not completely consistent across treatments. However, post-hoc Egg-vs.-J2 comparisons showed that this interaction was driven primarily by the nematode control, while none of the *C. rosea* treatments differed significantly in root fresh weight between inoculation types. Thus, this interaction does not provide strong evidence for isolate-specific differences in root biomass responses to egg versus J2 inoculation. Taken together, the biomass data suggest that the tested *C. rosea* isolates did not produce consistent growth-promoting or growth-inhibitory effects under the conditions tested, and that the differences observed between inoculation systems were largely restricted to specific treatment–inoculum combinations.

Overall, the results highlight substantial intraspecific variation in the biocontrol potential of *C. rosea* and indicate that the efficacy of individual isolates can vary with the nematode inoculations systems. This was most evident for root galling, where suppression differed substantially between egg- and J2-inoculated plants, whereas differences in shoot biomass were restricted to a subset of isolates and should be interpreted cautiously given the non-equivalent experimental conditions. The findings therefore emphasize the importance of considering nematode developmental stage and the experimental conditions associated with each inoculation approach when evaluating and selecting candidate isolates for biological control. Based on their overall galling-suppression profiles across the two inoculation methods, isolates 1308, 1910, 1923, 1952, 2084, 2135, and 2676 were selected for subsequent in vitro metabolite assays. These assays may help determine whether differences observed in planta are associated with direct nematicidal activity of fungal metabolites.

### 3.2. Activity of Culture Filtrates Reveals Predominantly Nematostatic Effects

The ability of sterile filtrates to delay M. incognita egg development indicates that soluble, fungus-derived factors can interfere with nematode development in the absence of direct hyphal contact. Previous studies have shown that *C. rosea* can produce extracellular lytic enzymes, including proteases, chitinases, and glucanases [12,18], as well as bioactive metabolites that may contribute to its antagonistic activity against nematodes [11,12,13,18,21,23]. The pronounced retention of individuals in earlier developmental stages observed for the more active isolates, particularly at higher filtrate concentrations and later observation times, may therefore result from the combined effects of multiple secreted compounds. However, the specific compounds and mechanisms responsible for the observed developmental inhibition were not investigated in the present study. Some isolate-concentration combinations also showed less pronounced effects at lower concentrations, indicating that the response was not uniformly concentration-dependent. Such patterns may reflect concentration-dependent biological responses to fungal metabolites, although the present data are insufficient to conclude that hormesis occurred [24,25]. Sublethal exposure to fungal metabolites may affect nematode development without causing immediate mortality [26,27], highlighting the need for further studies to identify the active compounds and clarify their effects on embryonic development and hatching.

Partial recovery of apparent J2 motility at some intermediate concentrations indicates that the response to *C. rosea* culture filtrates was not uniformly irreversible or concentration-dependent over time. Several mechanisms could potentially contribute to this pattern, including degradation or reduced stability of bioactive metabolites during incubation, changes in metabolite activity over time, or a transient physiological inhibition of a proportion of the J2 population. Similar patterns have been reported in filtrate-based nematode assays, where dilution, degradation of bioactive compounds, or physiological responses of nematode may contribute to variation in motility over time [26,28,29]. However, the present assay did not distinguish between these mechanisms, and therefore the observed recovery should be interpreted as a change in apparent motility rather than evidence of recovery from irreversible paralysis. Importantly, despite occasional increases in motility at intermediate concentrations, the overall suppressive effect of the filtrates increased with exposure time, and by day 7 all tested filtrate treatments showed significantly lower J2 activity than the nematode control after Holm adjustment. These findings suggest that culture filtrates can exert sustained effects on J2 motility, although the magnitude and temporal stability of these effects depend on isolate and concentration.

The moderate reduction in J2 infectivity following prolonged exposure to the highest filtrate concentration, together with the isolate- and concentration-dependent reductions in galling observed in planta, further indicates that strong inhibition of J2 motility does not necessarily translate directly into complete loss of infectivity or equivalent suppression of nematode development in plants. Although prolonged exposure to culture filtrates did not consistently prevent subsequent infection, significant reductions in galling were observed for several isolate-concentration combinations, particularly at 25%. These effects on disease development were accompanied by treatment-dependent variation in plant biomass, with significant differences detected in both shoot and root fresh weight; however, these responses did not consistently correspond to reductions in galling. Together, these findings indicate that the effects of culture filtrates on J2 infectivity can persist sufficiently to reduce disease development in some cases, but that their effects are strongly dependent on isolate, concentration, and biological endpoint. Thus, while *C. rosea* culture filtrates can strongly inhibit egg development and J2 motility, inhibition of motility does not uniformly translate into equivalent reductions in subsequent infectivity or in planta nematode suppression [14,21].

### 3.3. Greenhouse Performance and Implications for Biological Control

The limited efficacy observed in the small-scale greenhouse trial aligns with previous greenhouse and field studies on *C. rosea*, where reductions in galling and nematode densities were often modest, variable, or transient [12,14,30]. Several factors may contribute to the difference between the strong activity observed in vitro and the more limited effects observed in soil. *C. rosea* is known to produce a variety of bioactive enzymes and metabolites, but the persistence and bioavailability of these compounds in soil may be influenced by environmental conditions, including nutrient availability, interactions with native microbiota, adsorption to soil particles, and degradation [22,31]. Unlike the controlled conditions of the sterile filtrate assays, compounds produced or released in soil may also be subjected to dilution and spatial heterogeneity, potentially reducing their effective concentrations. A second possible explanation is the establishment of the fungus in the rhizosphere. Spore-based applications depend on successful fungal establishment and persistence in the soil and near the roots, and differences in colonization could influence the duration and magnitude of biocontrol activity [2,13]. In the present study, however, *C. rosea* establishment and root or rhizosphere colonization were not quantified. Therefore, differences in fungal establishment or colonization relative to nematode infection dynamics remain hypotheses that require experimental verification. Similar temporal patterns, in which early reductions in galling are not maintained throughout the season, have been reported for *C. rosea* PHP1701, *Purpureocillium lilacinum*, and *Pochonia chlamydosporia* [14,32,33,34,35].

The absence of significant effects on egg mass production, total egg counts, and eggs per egg mass further indicates that the treatments did not substantially disrupt nematode reproduction under the conditions of the small-scale greenhouse trial. This contrasts with the pronounced effects observed in vitro and may reflect differences in exposure conditions, compound availability, or the ability of the fungus and its metabolites to reach developing nematodes or eggs within the plant–soil system. The transient nature of some effects observed in other biocontrol studies, together with the recovery of J2 motility and retention of infectivity following metabolite exposure in vitro, suggests that sustained exposure may be important for achieving consistent suppression in planta.

Importantly, no clear evidence of phytotoxic effects was observed, although root fresh weight differed significantly among treatments overall. However, none of the individual *C. rosea* isolates differed significantly from the nematode control following Tukey adjustment. Thus, the observed variation in root biomass was not attributable to a significant reduction caused by any of the tested isolates. This aligns with reports describing *C. rosea* as a benign rhizosphere inhabitant that may indirectly enhance plant performance through reduced pathogen pressure or improved rhizosphere functioning [2,36,37].

The large-scale greenhouse trial nevertheless demonstrated that selected isolates could provide biologically meaningful suppression during the early stages of infection. The early-season performance of these isolates was broadly comparable to PHP1701, which similarly reduces initial galling and delays nematode reproduction on tomato roots [14]. Notably, isolate 2084 achieved stronger early gall suppression than PHP1701, highlighting the potential of different isolates to match or surpass formulated isolates.

The disappearance of treatment differences in galling later in the season may reflect the increasing nematode population under continuous host availability and favorable greenhouse conditions. Importantly, vegetative growth increased similarly across treatments, with no significant Treatment × Date interaction, indicating that the temporal pattern in gall suppression was not accompanied by detectable treatment-specific differences in plant growth. However, changes in fungal abundance, activity or persistence were not measured, and therefore the mechanisms underlying the decline in treatment efficacy cannot be determined from the present study. Similar temporal patterns have been reported for *C. rosea* PHP1701 [14], *P. chlamydosporia* [38], and *P. lilacinum* [39], emphasizing the potential importance of application timing and successful fungal establishment.

Future studies should therefore assess fungal establishment and persistence in the rhizosphere, for example through re-isolation, isolate-specific qPCR, or microscopy, together with measurements of fungal abundance and activity over time. Such approaches would help determine whether differences in colonization, persistence, or metabolite activity contribute to the temporal variation in biocontrol efficacy observed here.

Although juvenile densities were numerically lower in some *C. rosea*-treated soils toward the end of the experiment, particularly following treatment with isolate 2084, these differences were not statistically significant. Thus, the observed patterns do not provide conclusive evidence for an effect on nematode development or reproduction. Nevertheless, the numerical differences observed at the final assessment may warrant further investigation, particularly in experiments designed to distinguish effects on nematode survival, development, and reproduction [13,14,40].

Perhaps the most agronomically relevant finding was that fungal treatments were associated with harvest-specific increases in individual fruit weight despite the eventual convergence in galling severity. Cumulative fruit number and fruit weight did not differ significantly among treatments, indicating that the observed differences in fruit size did not translate into a statistically significant increase in total yield. Nevertheless, isolates 1308 and 2084 produced heavier fruits than the nematode control at multiple harvest stages, while isolates 1952 and 1910 showed significant increases at individual later harvests. These results suggest that early or transient suppression of nematode infection may contribute to maintaining plant performance, although the present data do not demonstrate a significant overall yield benefit. Similar temporal patterns of early nematode suppression followed by limited long-term population control have been reported for PHP1701, where early suppression was associated with yield related benefits [14].

Overall, our results indicate that *C. rosea* provides temporally limited yet biologically meaningful suppression of *M. incognita*. Early- and mid-season reductions in galling, delayed effects on soil juvenile populations, and harvest-specific increases in individual fruit weight highlight its potential as a component of integrated nematode management strategies [2]. Among the isolates tested, 1308, 1910, and particularly 2084 performed most consistently across multiple experimental scales, supporting their selection from the initial screening. Although complete nematode control was not achieved, and cumulative yield was not significantly increased, the occurrence of harvest-specific increases in fruit weight suggests that partial or transient suppression of nematode activity may contribute to maintaining crop performance.

From an agronomic perspective, these findings suggest that *C. rosea* could potentially contribute to integrated nematode management approaches. In the present study, early suppression was observed under greenhouse conditions at an initial nematode density of approximately 0.3 eggs/J2 per gram of soil. This value should not be considered an established action threshold, but rather a preliminary reference point derived from the conditions tested in this study and requiring validation across different nematode densities, cultivars and productions systems. Early suppression by selected isolates could contribute to reducing the impact of *M. incognita* during critical stages of crop development. However, whether such suppression could influence nematicide application timing or allow reduced application rates remain to be experimentally determined. The strong early activity of isolate 2084, which matched or exceeded PHP1701, further underscores the value of identifying isolates with superior biocontrol potential.

## 4. Materials and Methods

### 4.1. Nematode Rearing

A Mi-virulent *Meloidogyne incognita* population (Reichenau II) [41] was reared on three-week-old tomato plants (*Solanum lycopersicum* cv. Oskar, Bigler Samen AG, Steffisburg, Switzerland) under greenhouse conditions (25 °C day, 19 °C night; 60% relative humidity; 15/9 h light/dark cycle). Severely galled root systems were uprooted and gently washed free of a 1:3 soil:silver sand mixture [14].

Second stage juveniles (J2) were extracted using a mist chamber. Eggs were isolated by cutting roots into 1 cm segments, shaking vigorously in 1% NaOCl (Dr. Grogg Chemie AG, Stettlen, Switzerland) for 3 min, and collecting them on a 20 μm sieve. Egg and J2 densities were determined using an inverted light microscope (Zeiss, Oberkochen, Germany, 5× magnification). During counting, J2 viability and motility were checked to ensure that viable and motile juveniles were used for inoculation. Egg and J2 suspensions were stored at 4 °C for no longer than 5 days before use [14].

Nematode identity and purity were verified periodically via DNA extraction from J2 and species-specific PCR, following Kiewnick et al. [42].

### 4.2. Clonostachys Collection and Culture Preparation

*Clonostachys rosea* isolates were obtained from the Agroscope Mycoscope database [43] (Supplementary Table S1). Isolates were transferred to potato dextrose agar (PDA VWR, Llinars del Vallès, Spain, 100 × 15 mm) plates supplemented with ampicillin (Thermo Fisher Diagnostics GMBH, Hennigsdorf, Germany, 100 µg mL^−1^) and erythromycin (50 µg mL^−1^) and incubated at 24 °C in darkness. Plates were subcultured on fresh PDA as required, to maintain active growth.

### 4.3. Stepwise Evaluation of Clonostachys rosea Biocontrol Potential

A stepwise screening strategy was used, with cucumber serving as the initial host for comparative isolate screening and tomato used for subsequent greenhouse evaluation of selected isolates under more complex growing conditions.

#### 4.3.1. Initial in Planta Screening on Cucumber

An initial in planta screening was conducted on cucumber (*Cucumis sativus* cv. Sprinter F1, Bigler Samen AG, Steffisburg, Switzerland) to compare the biocontrol activity of the *C. rosea* isolate collection against *M. incognita* eggs and J2. The *C. rosea* strains (Supplementary Table S1; Supplementary Figure S1) were evaluated for their ability to suppress nematode infection. *C. rosea* strain PHP1701 (Andermatt Biocontrol Suisse, Grossdietwil, Switzerland; [14]) served as a positive control.

Pots (25 mL) were filled to one-quarter of their volume with steamed potting soil and inoculated with 300 eggs or 250 J2. For fungal treatments a 1 × 1 cm PDA agar block from a seven-day-old fungal culture was placed on the soil surface, whereas untreated control pots received a 1 × 1 cm sterile PDA block. The pots were then filled three-quarters of their volume with steamed potting soil and maintained at 24 °C for 7 days under moist conditions to allow fungal establishment. Pre-germinated cucumber seedlings were then transplanted. Plants were grown for 28 days (J2 treatments) or 35 days (eggs treatments) under 16/8 h day/night cycles at 24 °C and 60% relative humidity. For J2-inoculated plants, root galling was evaluated after 28 days, consistent with the 21–28-day evaluation period routinely used in our laboratory, during which galls become clearly visible for reliable scoring. For egg-inoculated plants, the assessment was extended to 35 days to allow sufficient time for egg development and subsequent infection by J2. In a preliminary egg-development assessment, egg development progressed from the egg stage through the pretzel stage to J2, with 78.8% ± 2.12% of the initially inoculated 300 eggs reaching the J2 stage after 14 days (Supplementary Table S12). Each treatment was replicated five times (*n* = 5).

At harvest, roots were gently washed free of soil, and root galling was visually scored for each plant using a 0–10 gall index described by Zeck (GI) [44], where 0 represents no galling and 10 represents the highest level of galling. The Gi for each treatment was calculated as the arithmetic mean of the individual plant scores. Shoot and root fresh weights were recorded. Strains causing a significant GI reduction compared with untreated controls were selected for further assays.

#### 4.3.2. In Vitro Culture Filtrate Assays

Selected *Clonostachys rosea* isolates [PHP1701 (positive control), 1308, 1910, 1923, 1952, 2084, 2135, and 2676] were cultured in half-strength potato dextrose broth (PDB VWR, Llinars del Vallès, Spain) under agitation (120 rpm, 25 °C) for 28 days. Cultures were filtered through sterile gauze, centrifuged (1000 rpm for 25 min), and sterile-filtered (0.22 µm).

Filtrates were tested at 0.5%, 1%, 5%, 10% and 25% (*v*/*v*) for effects on J2 motility, and egg development. For each treatment, 250 J2 and 350 eggs were exposed to the respective filtrate concentration. Observations were made at 1, 2, 3 and 7 days post-treatment for J2 and 1, 3, 6 and 9 days for eggs. A visual evaluation of J2 motility and fitness after incubation was assessed as described by Oldani et al. [45]. Briefly, J2 were counted (*n* = 4) on a nematode counting slide using an inverted light microscope (Zeiss, 5× magnification) and classified as: active, showing normal motile behavior comparable to water control; inhibited, showing twisted or coiled shapes, broken or irregular movement; or immobile, showing no visible movement and a straightened body. Egg development (*n* = 4) was classified into three morphological categories: egg, pretzel, and J2. All developmental stages prior to the appearance of the characteristic pretzel-shaped embryo were classified as “egg”. Embryos showing the characteristic pretzel shape were classified as “pretzel”, while fully developed J2 were classified as “J2”. Sterile water was used as the negative control.

After 7 days, all J2 from each treatment were inoculated onto cucumber seedlings. Root galling and plant growth parameters were evaluated after 28 days to assess the effects of filtrate exposure on J2 infectivity. GI was scored as described above [44]. Only strains showing significant nematode suppression were advanced to greenhouse testing.

#### 4.3.3. Greenhouse Evaluation on Tomato

Small-scale trials: Thirteen-centimeter pots were filled with 500 cm^3^ of a 1:3 soil:sand mixture, inoculated with 6000 J2 and treated with a suspension of *C. rosea* conidia (1 × 10^7^ spores mL^−1^). The pot volume was completed with an additional 200 cm^3^ of soil:sand mixture and kept moist for 7–10 days to allow fungal establishment before transplanting two-week-old tomato seedlings (*Solanum lycopersicum* cv. Oskar). Each treatment was replicated eight times (*n* = 8). After 5–6 weeks, GI (scored as described above [44]), egg mass counts, and root fresh weights were recorded.

Large-Scale Trial: Fifteen-liter pots were filled with heat-sterilized field soil, inoculated with *M. incognita* (5000 nematodes per pot: 46% eggs, 54% J2) or left nematode-free as controls. Selected *C. rosea* isolates were grown on PDB at 24 °C for 21 days in darkness under continuous shaking at 120 rpm. Conidia were harvested and quantified using a modified Neubauer chamber (Blaubrand^®^, Brand GmbH + Co KG, Wertheim, Germany). Each fungal treatment was initially applied at 1 × 10^7^ conidia in 200 mL of water, three days after nematode inoculation, and subsequently reapplied at monthly intervals throughout the experiment. Nematode inoculated pots receiving an equal volume of distilled water instead of fungal inoculum served as untreated controls. Each treatment was replicated sixteen times.

Tomato rootstocks (cv. Roterno F1 RZ grafted onto Maxifort, De Ruiter Seeds, Bergschenhoek, The Netherlands) were transplanted 7 days after the initial fungal application. Maxifort is a commonly used tomato rootstock in Switzerland and carries *Mi* resistance against *Meloidogyne* spp. However, the *M. incognita* population used in this study (Reichenau II) has previously been reported to overcome this resistance [41]. Plants were irrigated and fertilized weekly with a soluble NPK fertilizer (Kristalon Red Acid, Yara, UK) via a drip irrigation system.

Root galling and plant growth parameters, specifically the length of the left and right branches, were assessed destructively at weeks 6, 10, 14 and 18, corresponding to the early, early-mid, mid-late, and late growth stages, respectively. At each sampling time, four plants per treatment were removed from the experiment (*n* = 4). Different plants were used at each time point, resulting in 16 plants sampled per treatment over the four assessment dates. For plant growth parameters, the lengths of the left and right branches were measured before the above-ground parts were removed to facilitate root assessment. Root systems were carefully uprooted and washed free of soil before root galling was visually scored for each plant using the 0–10 scale described above [44].

Fruit harvesting began after the first destructive sampling at week 6. Ripe fruits were subsequently harvested weekly from the plants remaining in the experiment, counted, and weighed. The following yield parameters were determined: mean number of fruits per harvest, mean fruit weight per harvest, mean number of fruits per plant, mean fruit weight per plant, and mean individual fruit weight. Yield increase was calculated as the percentage increase in mean total fruit weight per plant relative to the untreated control.

### 4.4. Statistical Analysis

All statistical analyses were performed in R version 4.4.2 [46]. Statistical methods were selected according to the distribution and scale of each response variable. Unless otherwise stated, statistical significance was assessed at *p* < 0.05. Data are presented as means ± standard error (SE).

#### 4.4.1. Statistical Analysis of Root Gall Index

Root gall index (GI), recorded on the ordinal 0–10 scale, was analyzed using non-parametric methods. For each inoculum type (eggs and second-stage juveniles [J2]), differences among *Clonostachys rosea* treatments were assessed using the Kruskal–Wallis test, followed by Dunn’s multiple-comparison test with Holm adjustment for pairwise comparisons. Adjusted *p*-values < 0.05 were considered statistically significant. GI data are presented as mean ± standard error (SE), together with median and interquartile range (IQR).

To assess whether the response to inoculum type differed among treatments, an ordinal logistic regression model was fitted with GI as the ordinal response and Treatment, Inoculum (egg or J2), and their interaction as fixed effects. The Treatment × Inoculum interaction was assessed using a likelihood-ratio test. Within each treatment, model-based contrasts with 95% confidence intervals between egg and J2 inoculation were subsequently estimated, with *p*-values adjusted for multiple comparisons using the Holm method. Adjusted *p*-values < 0.05 were considered statistically significant.

#### 4.4.2. Statistical Analysis of Biomass

Shoot and root fresh weight were analyzed using linear models with *C. rosea* treatment, inoculum type, and their interaction as fixed effects. Model residuals were assessed for normality using the Shapiro–Wilk test, and homogeneity of variances was evaluated using residual diagnostics. Where significant treatment effects were detected, estimated marginal means with 95% confidence intervals and Tukey-adjusted pairwise comparisons were used to assess differences among treatments. In addition, model-based contrasts were used to compare egg- and J2-inoculated plants within each treatment, with *p*-values adjusted using the Holm method. Biomass data are presented as means ± standard error (SE), and adjusted *p*-values < 0.05 were considered statistically significant.

#### 4.4.3. Statistical Analysis of Nematode Reproduction

Nematode reproduction parameters, including egg mass number, total egg count, and the number of eggs per egg mass, were analyzed separately using one-way analysis of variance (ANOVA), with *C. rosea* treatment as the fixed factor. Normality of model residuals was assessed using the Shapiro–Wilk test, and homogeneity of variances was assessed using Bartlett’s test. Where the overall treatment effect was significant, Tukey’s honestly significant difference (HSD) test was used for post-hoc pairwise comparisons. Reproduction data are presented as means ± standard error (SE), and *p*-values < 0.05 were considered statistically significant.

#### 4.4.4. Statistical Analysis of Fruit Production and Yield

Cumulative fruit number, cumulative fruit weight, and mean individual fruit weight per plant were analyzed separately using one-way ANOVA, with *C. rosea* treatment as the fixed factor. Model assumptions were assessed using the Shapiro–Wilk test for normality of residuals and residual diagnostics for homogeneity of variance. Where appropriate, treatment means were compared using Tukey’s HSD test. Estimated marginal means and 95% confidence intervals were calculated for model-based treatment comparisons.

Fruit number at individual harvests was analyzed using a generalized linear mixed-effects model with a negative binomial distribution to account for the count nature of the response and potential overdispersion. Treatment, harvest stage, and their interaction were included as fixed effects, with Plant included as a random effect to account for repeated observations of the same plants across harvests. Model fit and dispersion were assessed using simulated residual diagnostics. Estimated marginal means with 95% confidence intervals were calculated on the response scale for treatment comparisons.

Individual fruit weight at each harvest was calculated for each plant as total fruit weight divided by the corresponding number of fruits harvested. These data were analyzed using a linear mixed-effects model with Treatment, harvest stage, and their interaction as fixed effects and Plant as a random effect to account for repeated observations of the same plants across harvests. Residual normality was assessed using the Shapiro–Wilk test and residual diagnostics. Because the Treatment × harvest-stage interaction was significant, treatment effects were subsequently evaluated within each harvest stage using estimated marginal means with 95% confidence intervals. Each *C. rosea* treatment was compared with the nematode control using contrasts against the control, with Holm adjustment for multiple comparisons within each harvest stage. Adjusted *p*-values < 0.05 were considered statistically significant.

Cumulative yield data are presented as means ± SE. Harvest-specific fruit number and individual fruit weight are presented as means ± SE for each treatment and harvest stage.

#### 4.4.5. Statistical Analysis of Egg Development

Egg development was analyzed using the observed numbers of eggs, pretzels, and second-stage juveniles (J2) recorded for each biological replicate at each observation day. Analyses were performed on the underlying counts. Eggs were compared with non-eggs (pretzels + J2) using a binomial generalized linear model with *C. rosea* treatment, observation day, and their interaction as fixed effects. Treatment was defined by isolate and culture-filtrate concentration. Model fit was assessed using simulated residual diagnostics, including tests for dispersion and residual uniformity.

Estimated marginal means with 95% confidence intervals were calculated for each treatment within each observation day, and each isolate–concentration treatment was compared with the nematode control using model-based contrasts. *p*-values were adjusted for multiple comparisons within each day using the Holm method. Adjusted *p*-values < 0.05 were considered statistically significant.

For descriptive presentation, the numbers of eggs, pretzels, and J2 were converted to percentages separately for each replicate using the total number of individuals counted in that replicate as the denominator. Data are presented as mean percentages ± standard error (SE) across biological replicates.

#### 4.4.6. Statistical Analysis of J2 Motility

J2 motility was analyzed using the observed numbers of active, inhibited, and immobile second-stage juveniles (J2) recorded for each biological replicate at each observation day. For statistical analysis, J2 were classified as active or non-active, with non-active J2 comprising inhibited and immobile individuals. Analyses were performed on the underlying counts rather than on percentages.

Because the initial binomial model showed separation associated with treatment combinations in which all observed J2 were non-active, bias-reduced logistic regression for binomial responses was used to obtain finite parameter estimates under separation. Separate models were fitted for each observation day, with the numbers of active and non-active J2 as the binomial response and Treatment as the fixed effect. Treatment was defined by isolate and culture-filtrate concentration, with the nematode control included as the reference treatment.

Model-based pairwise comparisons with 95% confidence intervals were used to compare each isolate–concentration treatment with the nematode control within each observation day. *p*-values were adjusted for multiple comparisons within each day using the Holm method. Adjusted *p*-values < 0.05 were considered statistically significant.

For descriptive presentation, the numbers of active, inhibited, and immobile J2 were converted to percentages separately for each biological replicate using the total number of individuals counted in that replicate as the denominator. Data are presented as mean percentages ± standard error (SE) across biological replicates.

## 5. Conclusions

This study demonstrates that the biocontrol potential of *Clonostachys rosea* against *Meloidogyne incognita* is strongly strain-dependent and influenced by nematode developmental stage, culture-filtrate concentration, and experimental context. Several isolates reduced root galling during the initial screening and showed pronounced inhibition of egg development and J2 motility in culture-filtrate assays. However, strong in-vitro activity did not consistently translate into sustained nematode suppression under greenhouse conditions, highlighting the influence of the plant–nematode–fungus environment on biocontrol efficacy. Selected isolates also produced early reductions in root galling and harvest-specific increases in individual fruit weight, whereas cumulative tomato fruit number and fruit weight were not significantly affected by treatment. Among the isolates tested, 1308, 1910, and 2084 showed comparatively strong activity across multiple experimental endpoints, although their effects varied with inoculation type, concentration, and assessment time. Overall, these findings indicate that *C. rosea* has potential as a component of integrated management of *M. incognita*, particularly through early suppression of nematode infection and development. However, reliable and sustained nematode control under soil and greenhouse conditions will require further optimization of isolate selection, application strategy, dosage, and fungal establishment.

## Data Availability

The authors confirm that the data supporting the results of this study are available in the article and its Supplementary Materials.

## Supplementary Materials

The following supporting information can be downloaded at https://www.mdpi.com/article/10.3390/plants15182809/s1, Figure S1: Morphological diversity of *Clonostachys rosea* strains obtained from the Agroscope Mycoscope database, evaluated for their biocontrol potential against *Meloidogyne incognita*. Images illustrate colony variation among strains; Figure S2: Shoot (A, B) and root (C, D) fresh weight of cucumber plants treated with *Clonostachys rosea* isolates and inoculated with *Meloidogyne incognita* eggs (A, C) or J2 (B, D). The negative control received nematode inoculation without fungal treatment, whereas PHP1701 served as the positive fungal control. Individual points represent individual plants; diamonds indicate treatment means and error bars represent ± SE; Figure S3: Antibiosis assay showing the effects of increasing concentrations (0.5, 1.0, 5.0, 10 or 25% *v*/*v*) of *Clonostachys rosea* culture filtrates from isolates 1923, 2135, and 2676 on *Meloidogyne incognita* egg development after 1, 3, 6, and 9 days of incubation. The nematode control is shown alongside each isolate for comparison. Data are presented as mean percentages ± SE of eggs, pretzels, and second-stage juveniles (J2) across biological replicates. Statistical comparisons of egg development between each isolate–concentration treatment and the nematode control were performed within each day using a binomial generalized linear model, with Holm adjustment for multiple comparisons. Asterisks indicate significant differences from the nematode control within the corresponding day (* *p* < 0.05); Figure S4: Antibiosis assay showing the effects of different concentrations (0.5, 1.0, 5.0, 10 or 25% *v*/*v*) of *Clonostachys rosea* culture filtrates from isolates 1923, 2135, and 2676 on *Meloidogyne incognita* J2 motility after 1, 3, 6, and 9 days of incubation. Points represent mean percentages of active J2 and error bars represent the standard error (SE) of replicates (*n* = 4). Connecting lines are included as visual guides between concentrations. The dashed horizontal line represents the corresponding nematode control. Statistical comparisons between each filtrate treatment and the nematode control were performed separately for each day using bias-reduced binomial logistic regression, with Holm adjustment for multiple comparisons. Adjusted *p* < 0.05 was considered statistically significant.; Figure S5: Effect of *Clonostachys rosea* spore suspension on tomato plants (*Solanum lycopersicum* cv. Oskar) in a small-scale trial. Gall index (A), root weight (B), egg mass (C), egg count (D), and eggs per egg mass (E) were recorded. Points represent individual replicates and error bars indicate standard errors. Gall index was analyzed using the Kruskal–Wallis test, whereas root fresh weight, egg mass, egg count, and eggs per egg mass were analyzed using one-way ANOVA. For root fresh weight, significant overall treatment effects were followed by Tukey’s post-hoc test. *p* < 0.05 was considered statistically significant; Table S1: *Clonostachys rosea* strains obtained from the Agroscope Mycoscope database and evaluated for their biocontrol potential against *Meloidogyne incognita*; Table S2: Root gall index (GI) of cucumber plants (*Cucumis sativus* cv. Sprinter F1) following treatment with *Clonostachys rosea* and inoculation with *Meloidogyne incognita* eggs or second-stage juveniles (J2); Table S3: Shoot fresh weight of cucumber plants (*Cucumis sativus* cv. Sprinter F1) following treatment with *Clonostachys rosea* strains and inoculation with *Meloidogyne incognita* eggs or second stage juveniles (J2); Table S4: Root fresh weight of cucumber plants (*Cucumis sativus* cv. Sprinter F1) following treatment with *Clonostachys rosea* strains and inoculation with *Meloidogyne incognita* eggs or second stage juveniles (J2); Table S5: In Vitro testing of *Clonostachys* rosea culture filtrate against *Meloidogyne incognita* egg development; Table S6: In Vitro testing of *Clonostachys rosea* culture filtrate against *Meloidogyne incognita* J2; Table S7: Gall index (GI) and plant biomass of cucumber plants 21 days after inoculation with *Meloidogyne incognita* J2 pre-exposed for seven days to culture filtrates of *Clonostachys rosea* isolates at different concentrations; Table S8: Small scale trial: gall index (GI), root biomass, and *Meloidogyne incognita* reproduction parameters following treatment with selected *Clonostachys rosea* isolates; Table S9: Root gall index (GI) of tomato plants (*Solanum lycopersicum* cv. Roterno F1 RZ grafted onto Maxiforf) following treatment with *Clonostachys rosea* and inoculation with *Meloidogyne incognita* in a large-scale trial; Table S10: Average branch length of tomato plants (*Solanum lycopersicum* cv. Roterno F1 RZ grafted onto Maxifort) following treatment with *Clonostachys rosea* and inoculation with *Meloidogyne incognita* in a large-scale trial; Table S11: Total number of second-stage juveniles recovered from soil in early (week 6), mid (week 10), and late (week 18) stages of tomato growth (*Solanum lycopersicum* cv. Roterno F1 RZ grafted onto Maxifort) following treatment with *Clonostachys rosea* and inoculation with *Meloidogyne incognita* in a large-scale trial; Table S12: Cumulative fruit production and fruit weight of tomato plants (*Solanum lycopersicum* cv. Roterno F1 RZ grafted onto Maxifort) following treatment with *Clonostachys rosea* selected isolates and inoculation with *Meloidogyne incognita* in a large-scale trial; Table S13: Fruit production and individual fruit weight at each harvest of tomato plants (*Solanum lycopersicum* cv. Roterno F1 RZ grafted onto Maxifort) treated with *Clonostachys rosea* selected isolates in a large-scale trial; Table S14: Development of *Meloidogyne incognita* eggs during incubation.
