# Supplementary material for: Targeting Meloidogyne incognita with Clonostachys rosea: Strain-Dependent Biocontrol Performance and Yield Effects Under Greenhouse Conditions
Source: Plants (Basel). 2026 Sep 13;15(18):2809. doi: 10.3390/plants15182809 (PMC13610539; doi:10.3390/plants15182809)
Supplement: Supplementary file 1 [file plants-15-02809-s001.zip › plants-4533369-supplementary.pdf]

Supplementary Figure S1. Morphological diversity of *Clonostachys rosea* strains obtained from the Agroscope Mycoscope database, evaluated for their biocontrol potential against *Meloidogyne incognita*. Images illustrate colony variation among strains.

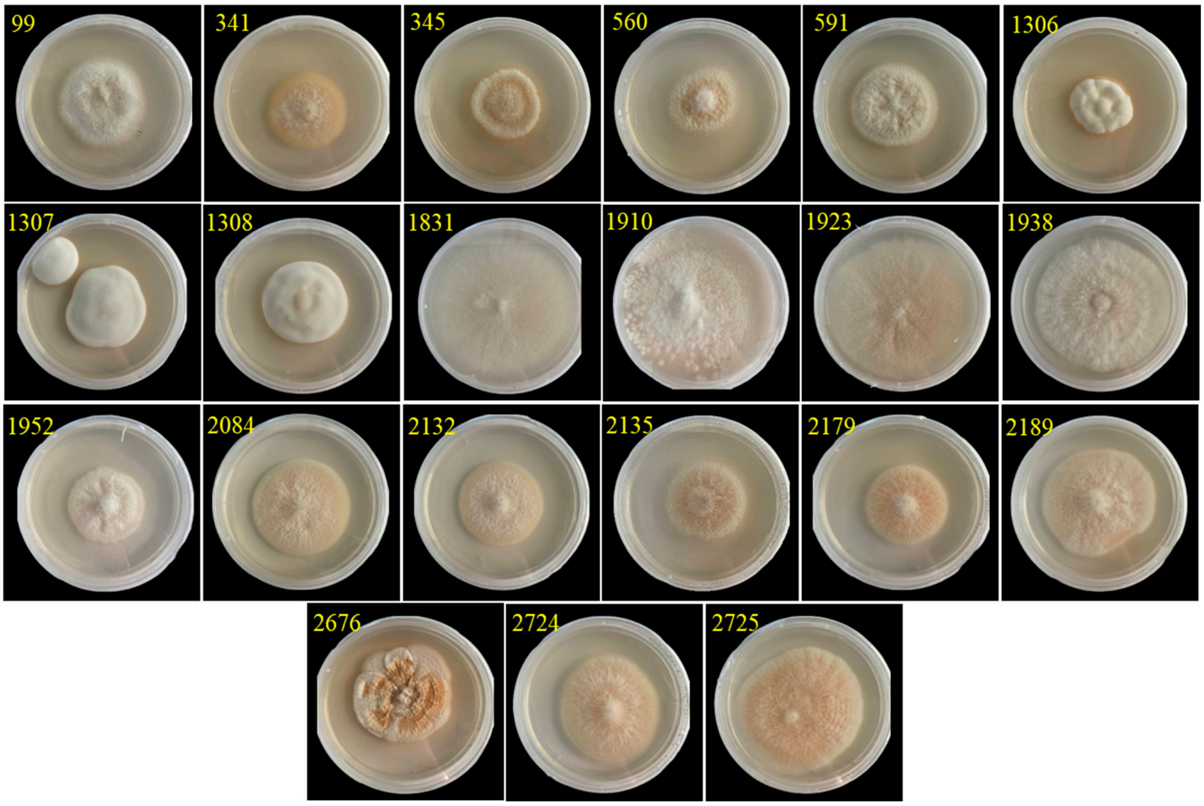

Supplementary Figure S2. Shoot (A, B) and root (C, D) fresh weight of cucumber plants treated with *Clonostachys rosea* strains and inoculated with *Meloidogyne incognita* eggs (A, C) or second-stage juveniles (J2; B, D). The negative control received nematode inoculation without fungal treatment, whereas PHP1701 served as the positive fungal control. Individual points represent individual plants; diamonds indicate treatment means and error bars represent ± SE.

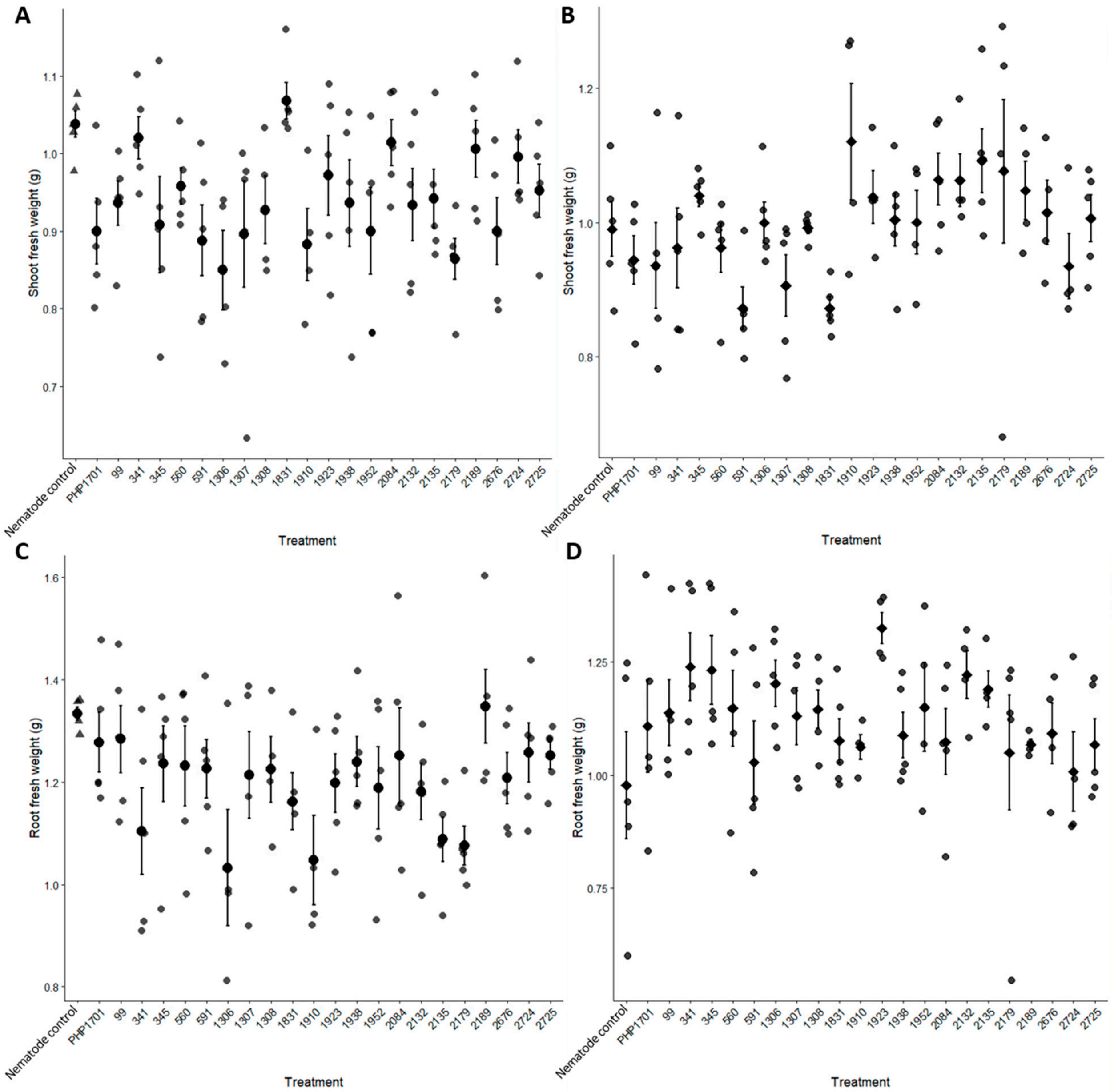

(A) Egg-inoculated shoot fresh weight: Shoot fresh weight differed significantly among treatments (one-way ANOVA, $F(22,89) = 1.87$, $p = 0.0213$); however, no significant pairwise differences were detected following Tukey's HSD adjustment (all adjusted $p > 0.05$); **(B) J2-inoculated shoot fresh weight:** Shoot fresh weight differed significantly among treatments (one-way ANOVA, $F(22,85) = 1.94$, $p = 0.0166$); however, no significant pairwise differences were detected following Tukey's HSD adjustment (all adjusted $P > 0.05$); **(C) Egg-inoculated root fresh weight:** Root fresh weight was not significantly affected by treatment (one-way ANOVA, $F(22,89) = 1.58$, $p = 0.0705$); **(D) J2-inoculated root fresh weight.** Root fresh weight was not significantly affected by treatment (one-way ANOVA, $F(22,84) = 1.19$, $p = 0.276$).

Supplementary Figure S3. Antibiosis assay showing the effects of increasing concentrations (0, 0.5, 1.0, 5.0, 10 or 25% media, v/v) of *Clonostachys rosea* culture filtrates from isolates 1923, 2135, and 2676 on *Meloidogyne incognita* egg development after 1, 3, 6, and 9 days of incubation. The nematode control is shown alongside each isolate for comparison. Data are presented as mean percentages ± SE of eggs, pretzels, and second-stage juveniles (J2) across biological replicates. Statistical comparisons of egg development between each isolate–concentration treatment and the nematode control were performed within each day using a binomial generalized linear model, with Holm adjustment for multiple comparisons. Asterisks indicate significant differences from the nematode control within the corresponding day (* $p < 0.05$).

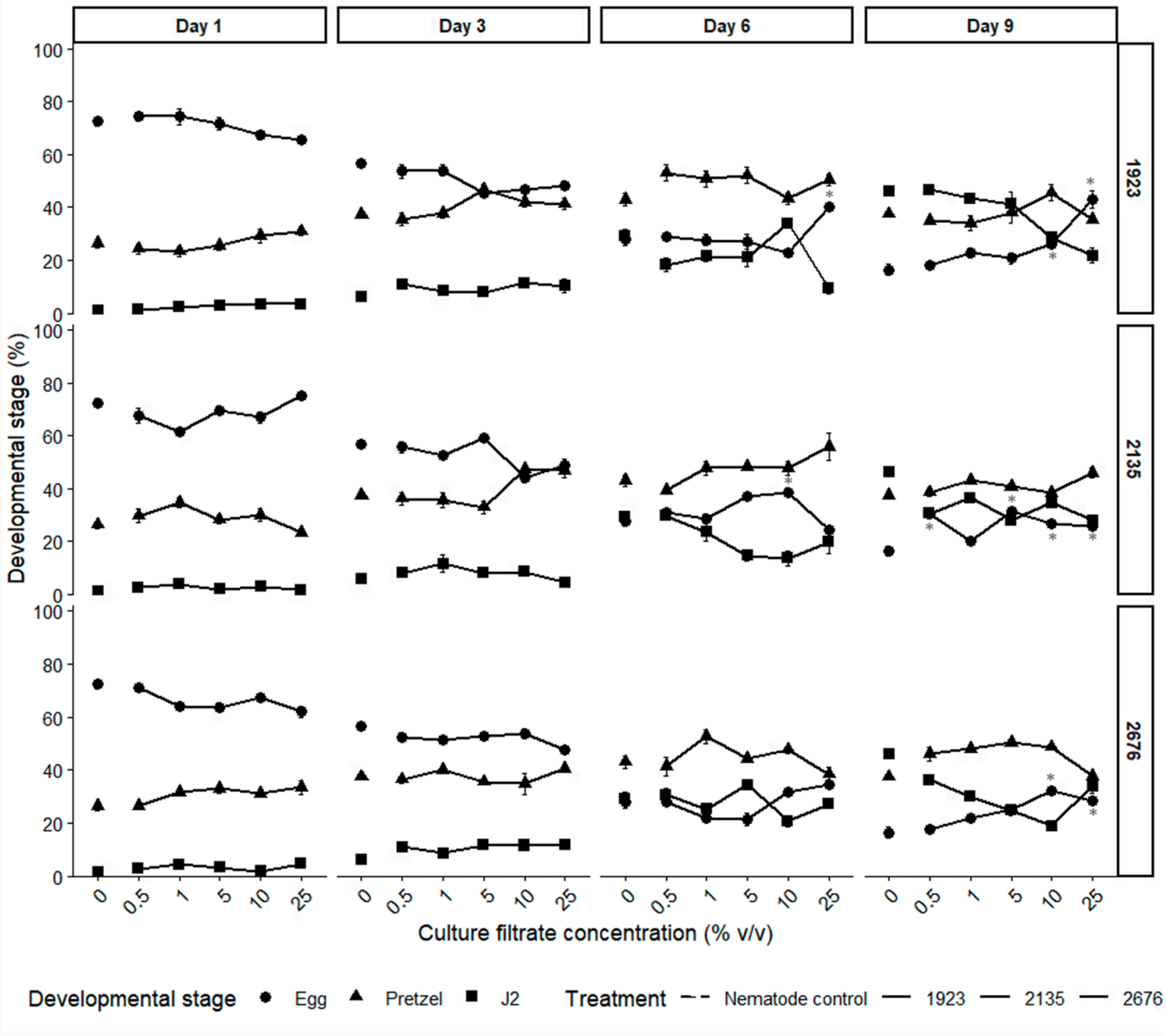

Supplementary Figure S4. Antibiosis assay showing the effects of different concentrations (0.5, 1.0, 5.0, 10 or 25% medium, v/v) of *Clonostachys rosea* culture filtrates from isolates 1923, 2135, and 2676 on *Meloidogyne incognita* J2 motility after 1, 2, 3 , and 7 days of incubation. Points represent mean percentages of active J2 and error bars represent the standard error (SE) of replicates (n = 4). Connecting lines are included as visual guides between concentrations. The dashed horizontal line represents the corresponding nematode control. Statistical comparisons between each filtrate treatment and the nematode control were performed separately for each day using bias-reduced binomial logistic regression, with Holm adjustment for multiple comparisons. Adjusted $p < 0.05$ was considered statistically significant.

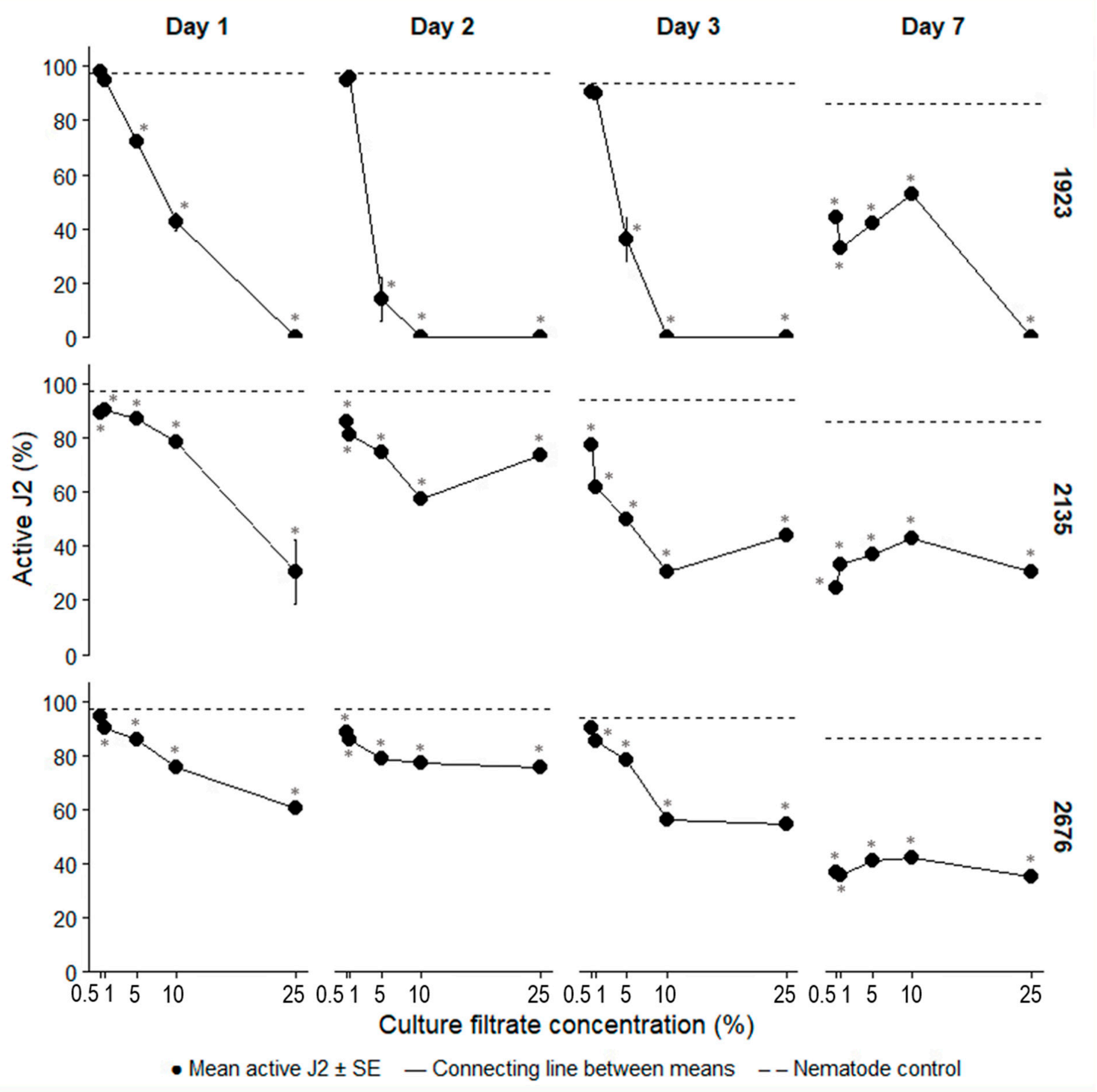

Supplementary Figure S5. Effect of *Clonostachys rosea* spore suspension on tomato plants (*Solanum lycopersicum* cv. Oskar) in a small-scale trial. Gall index (A), root weight (B), egg mass (C), egg count (D), and eggs per egg mass (E) were recorded. Points represent individual replicates and error bars indicate standard errors. Gall index was analyzed using the Kruskal–Wallis test, whereas root fresh weight, egg mass, egg count, and eggs per egg mass were analyzed using one-way ANOVA. For root fresh weight, significant overall treatment effects were followed by Tukey's post-hoc test. $P < 0.05$ was considered statistically significant.

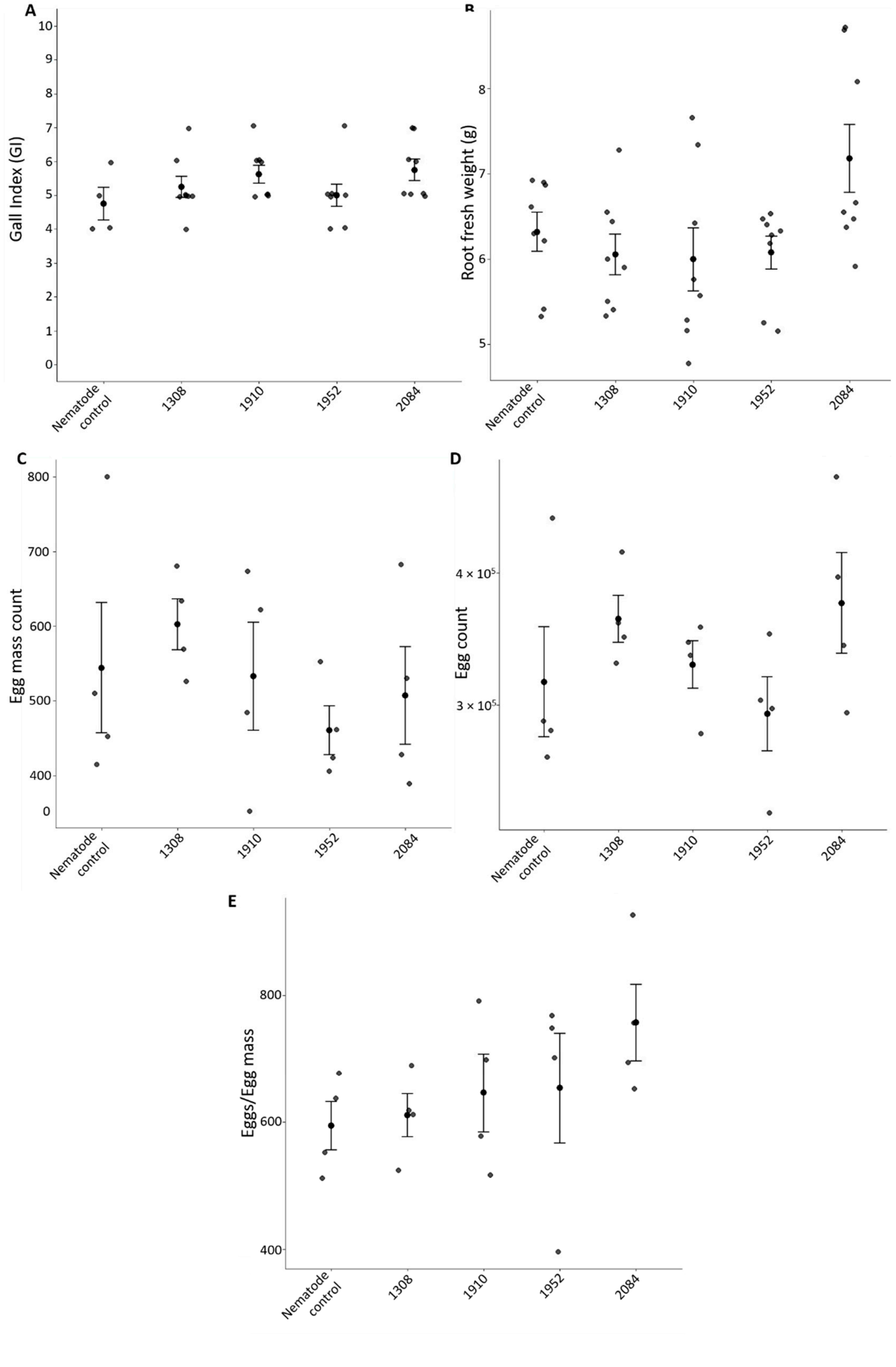

Supplementary Table S1. *Clonostachys rosea* strains obtained from the Agroscope Mycoscope database and evaluated for their biocontrol potential against *Meloidogyne incognita*.

Accecion number	Collection site	Taxonomy remarks	Host/Substrate	Date of sampling	Molecular sequence
99	-	*Clonostachys rosea* (telem. *Bionectria ochroleuca*)	Vine	2024	Yes
341	Switzerland	*Clonostachys* sp. (*Gliocladium roseum*)	Spring barley cv. Célinka	2012	Yes
345	Switzerland	*Gliocladium roseum* (*Clonostachys rosea*)	Spring barley cv. Célinka	2012	No
560	Switzerland	*Clonostachys rosea* (= *Bionectria ochroleuca*)	-	1977	Yes
591	Switzerland	*Clonostachys* sp.	White lupine	2001	Yes
1306	Switzerland	*Clonostachys* sp.	Rainwater	2016	Yes
1307	Switzerland	*Clonostachys* sp.	Rainwater	2016	Yes
1308	Switzerland	*Clonostachys* sp.	Rainwater	2016	Yes
1831	Switzerland	*Bionectria ochroleuca*	Avocado	2019	Yes
1910	Guyana	-	-	2018	Yes
1923	Guyana	-	*Astrocaryum paramaca*	2018	Yes
1938	Guyana	-	*Astrocaryum paramaca*	2018	Yes
1952	Guyana	-	*Astrocaryum paramaca*	2018	Yes
2084	Switzerland	*Clonostachys rosea*	Nursery/ Rootstock wood	2019	Yes
2132	Switzerland	*Clonostachys rosea*	Nursery/ Rootstock wood	2019	Yes
2135	Switzerland	*Clonostachys rosea*	Nursery/ Rootstock wood	2019	Yes
2179	Switzerland	*Clonostachys rosea*	Nursery/ Rootstock wood	2019	Yes
2189	Switzerland	*Clonostachys rosea*	Nursery/ Rootstock wood	2019	Yes
2676	Switzerland	*Clonostachys rosea*	Barbus wood	2017	Yes
2724	Switzerland	*Clonostachys rosea*	Apple tree (roots)	2018	Yes
2725	Switzerland	*Clonostachys rosea*	Apple tree (roots)	2018	Yes

Supplementary Table S2 Root gall index (GI) of cucumber plants (*Cucumis sativus* cv. Sprinter F1) following treatment with *Clonostachys rosea* and inoculation with *Meloidogyne incognita* eggs or second-stage juveniles (J2).

Treatment	Egg GI (mean ± SE)	Egg Median (IQR)	Egg Dunn-Holm group	J2 GI (mean ± SE)	J2 Median (IQR)	J2 Dunn-Holm group	Egg vs J2 *P*adj
**Nematode control**	7.8 ± 0.20	8.0 (0.0)	b	6.8 ± 0.20	7.0 (0.0)	b	0.0804
**PHP1701**	6.8 ± 0.20	7.0 (0.0)	ab	6.4 ± 0.24	6.0 (1.0)	abc	1.0000
**99**	6.2 ± 0.37	6.0 (1.0)	ab	6.6 ± 0.24	7.0 (1.0)	ab	0.3374
**341**	5.2 ± 0.49	6.0 (2.0)	a	6.2 ± 0.20	6.0 (0.0)	abcd	1.0000
**345**	6.6 ± 0.24	7.0 (1.0)	ab	4.6 ± 0.24	5.0 (1.0)	abcde	1.0000
**560**	7.4 ± 0.24	7.0 (1.0)	ab	6.4 ± 0.24	6.0 (1.0)	abc	2.99×10^{-7}
**591**	6.8 ± 0.37	7.0 (1.0)	ab	3.6 ± 0.24	4.0 (1.0)	cde	0.1832
**1306**	7.0 ± 0.41	7.0 (0.5)	ab	3.8 ± 0.37	4.0 (1.0)	acde	1.0000
**1307**	7.2 ± 0.37	7.0 (1.0)	ab	5.0 ± 0.89	5.0 (1.0)	abcde	0.0795
**1308**	7.5 ± 0.29	7.5 (1.0)	ab	3.4 ± 0.24	3.0 (1.0)	de	5.79×10^{-9}
**1831**	5.8 ± 0.37	6.0 (1.0)	ab	3.2 ± 0.20	3.0 (0.0)	e	0.8103
**1910**	6.0 ± 0.41	6.0 (0.5)	ab	5.2 ± 0.37	5.0 (1.0)	abcde	2.67×10^{-4}
**1923**	6.6 ± 0.24	7.0 (1.0)	ab	3.8 ± 0.37	4.0 (1.0)	acde	2.99×10^{-7}
**1938**	5.4 ± 0.40	6.0 (1.0)	a	5.2 ± 0.20	5.0 (0.0)	abcde	1.0000
**1952**	7.0 ± 0.00	7.0 (0.0)	ab	5.2 ± 0.20	5.0 (0.0)	abcde	3.33×10^{-11}
**2084**	5.2 ± 0.37	5.0 (1.0)	a	4.2 ± 0.20	4.0 (0.0)	abcde	0.0021
**2132**	6.6 ± 0.24	7.0 (1.0)	ab	4.8 ± 0.37	5.0 (1.0)	abcde	0.0157
**2135**	5.4 ± 0.40	6.0 (1.0)	a	5.4 ± 0.24	5.0 (1.0)	abcde	0.0157
**2179**	5.8 ± 0.20	6.0 (0.0)	ab	5.6 ± 0.24	6.0 (1.0)	abcde	0.8407
**2189**	7.0 ± 0.32	7.0 (0.0)	ab	6.4 ± 0.24	6.0 (1.0)	abc	4.24×10^{-4}
**2676**	6.2 ± 0.37	6.0 (1.0)	ab	5.0 ± 0.32	5.0 (0.0)	abcde	4.24×10^{-4}
**2724**	7.0 ± 0.32	7.0 (0.0)	ab	5.8 ± 0.37	6.0 (1.0)	abcde	4.24×10^{-5}
**2725**	6.8 ± 0.37	7.0 (1.0)	ab	5.4 ± 0.24	5.0 (1.0)	abcde	0.0157

Values are presented as mean GI ± SE and median (IQR). For each inoculum type, differences among treatments were assessed using the Kruskal–Wallis test followed by Dunn's multiple-comparison test with Holm adjustment. Different letters indicate significant differences among treatments within the respective inoculum type ($P < 0.05$). The nematode control consisted of plants inoculated with *M. incognita* but not treated with *C. rosea*, whereas PHP1701 was included as the positive fungal control. Egg and J2 responses were additionally compared within each treatment using an ordinal logistic regression model including Treatment, Inoculum, and their interaction. Pairwise contrasts between inoculum types were adjusted for multiple testing across treatments using the Holm method; significant contrasts are indicated by *P*adj < 0.05.

Supplementary Table S3. Shoot fresh weight of cucumber plants (*Cucumis sativus* cv. Sprinter F1) following treatment with *Clonostachys rosea* strains and inoculation with *Meloidogyne incognita* eggs or second stage juveniles (J2).

Treatment	Shoot fresh weight (g)		Egg vs J2 Holm-adjusted *P*
	Egg[1]	J2[2]	
**Nematode Control**	1.04 ± 0.02[a]	0.99 ± 0.04[a]	0.4454
**PHP1701**	0.90 ± 0.04[a]	0.94 ± 0.04[a]	0.4842
**99**	0.94 ± 0.03[a]	0.94 ± 0.06[a]	1.0000
**341**	1.02 ± 0.03[a]	0.96 ± 0.06[a]	0.3567
**345**	0.91 ± 0.06[a]	1.04 ± 0.02[a]	0.0369 *
**560**	0.96 ± 0.02[a]	0.96 ± 0.04[a]	0.9493
**591**	0.89 ± 0.05[a]	0.87 ± 0.03[a]	0.7991
**1306**	0.85 ± 0.05[a]	1.00 ± 0.03[a]	0.0255 *
**1307**	0.90± 0.07[a]	0.91 ± 0.04[a]	0.8736
**1308**	0.93 ± 0.04[a]	0.99 ± 0.01[a]	0.3339
**1831**	1.07 ± 0.02	0.87 ± 0.02[a]	0.0081 *
**1910**	0.88 ± 0.05[a]	1.12 ± 0.09[a]	0.0009 *
**1923**	0.97 ± 0.05[a]	1.04 ± 0.04[a]	0.3265
**1938**	0.94 ± 0.06[a]	1.00 ± 0.04[a]	0.2801
**1952**	0.90 ± 0.06[a]	1.00 ± 0.05[a]	0.1348
**2084**	1.01 ± 0.03[a]	1.06 ± 0.04[a]	0.4267
**2132**	0.93 ± 0.05[a]	1.06 ± 0.04[a]	0.0552
**2135**	0.94 ± 0.04[a]	1.09 ± 0.05[a]	0.0179 *
**2179**	0.86 ± 0.03[a]	1.08 ± 0.11[a]	0.0009 *
**2189**	1.01 ± 0.04[a]	1.05 ± 0.04[a]	0.5338
**2676**	0.90 ± 0.04[a]	1.02 ± 0.05[a]	0.0858
**2724**	1.00 ± 0.03[a]	0.94 ± 0.05[a]	0.3607
**2725**	0.95 ± 0.03[a]	1.01 ± 0.03[a]	0.3907

Values represent mean ± SE. Lowercase letters indicate Tukey's HSD grouping for comparisons among treatments within each inoculation type ($P < 0.05$). Egg vs J2 comparisons were performed within each treatment, with *P*-values adjusted using the Holm method. Bold *P*-values indicate significant differences between inoculation types ($P < 0.05$).

Supplementary Table S4. Root fresh weight of cucumber plants (*Cucumis sativus* cv. Sprinter F1) following treatment with *Clonostachys rosea* strains and inoculation with *Meloidogyne incognita* eggs or second stage juveniles (J2).

**Treatment**	**Root frsh weight (g)**		**Egg vs J2 Holm-adjusted *P***
	**Egg**[1]	**J2**[2]	
**Nematode control**	1.33 ± 0.01^{a}	0.98 ± 0.12	0.0083*
**PHP1701**	1.28 ± 0.06^{a}	1.11 ± 0.10	0.0841
**99**	1.28 ± 0.07^{a}	1.14 ± 0.07	0.1375
**341**	1.10 ± 0.08^{a}	1.24 ± 0.08	0.1664
**345**	1.24 ± 0.07^{a}	1.23 ± 0.08	0.9674
**560**	1.23 ± 0.08^{a}	1.15 ± 0.08	0.3919
**591**	1.23 ± 0.06^{a}	1.03 ± 0.09	0.8915
**1306**	1.03 ± 0.11^{a}	1.20 ± 0.05	0.1043
**1307**	1.21 ± 0.08^{a}	1.13 ± 0.06	0.3919
**1308**	1.23 ± 0.06^{a}	1.15 ± 0.04	0.4476
**1831**	1.16 ± 0.06^{a}	1.08± 0.05	0.3807
**1910**	1.05 ± 0.09^{a}	1.06 ± 0.03	0.8911
**1923**	1.20 ± 0.06^{a}	1.33 ± 0.03	0.2228
**1938**	1.24 ± 0.04^{a}	1.09 ± 0.05	0.1222
**1952**	1.19 ± 0.08^{a}	1.15 ± 0.10	0.7147
**2084**	1.25 ± 0.09^{a}	1.07 ± 0.07	0.0706
**2132**	1.18 ± 0.06^{a}	1.22 ± 0.05	0.6969
**2135**	1.09 ± 0.04^{a}	1.14 ± 0.06	0.6244
**2179**	1.08 ± 0.04^{a}	1.05 ± 0.13	0.7908
**2189**	1.35 ± 0.07^{a}	1.07 ± 0.01	0.1665
**2676**	1.21 ± 0.05^{a}	1.09 ± 0.07	0.2674
**2724**	1.26 ± 0.05^{a}	1.01 ± 0.09	0.3537
**2725**	1.25 ± 0.03^{a}	1.07 ± 0.06	0.0618

Values represent mean ± SE. Lowercase letters indicate Tukey's HSD grouping for comparisons among treatments within each inoculation type ($P < 0.05$). Egg vs J2 comparisons were performed within each treatment, with *P*-values adjusted using the Holm method. Bold *P*-values indicate significant differences between inoculation types ($P < 0.05$).

Supplementary Table S5. In Vitro testing of *Clonostachys* rosea culture filtrate against *Meloidogyne incognita* egg development.

Treatment	Concentration	Day 1			Day 3			Day 6			Day 9		
		Developmental stage (%)			Developmental stage (%)			Developmental stage (%)			Developmental stage (%)		
	(media %, v/v)	Egg[1]	Pretzel[2]	J2[3]	Egg[4]	Pretzel[5]	J2[6]	Egg[7]	Pretzel[8]	J2[9]	Egg[10]	Pretzel[11]	J2[12]
Control	0	72.4 ± 1.69	26.4 ± 1.54	1.2 ± 0.37	56.7 ± 0.33	37.3 ± 0.88	6.0 ± 1.00	27.9 ± 2.14	43.0 ± 2.20	29.2 ± 2.32	16.4 ± 1.99	37.4 ± 0.93	46.2 ± 1.80
PHP1710	0.5	72.0 ± 1.73	26.3 ± 1.45	1.7 ± 0.33	52.8 ± 2.95	41.2 ± 2.06	6.0 ± 1.35	28.9 ± 5.05	45.5 ± 4.75	25.6 ± 0.30	25.0 ± 1.08*	38.0 ± 1.08	37.0 ± 1.87
	1	64.3 ± 1.67	34.7 ± 1.20	1.0 ± 0.58	49.8 ± 1.11	42.5 ± 2.02	7.8 ± 1.70	31.0 ± 1.15	49.7 ± 1.45	19.3 ± 0.33	29.0 ± 2.68***	34.5 ± 3.59	36.5 ± 1.32
	5	64.4 ± 0.30	34.9 ± 0.10	0.7 ± 0.34	51.8 ± 2.56	36.8 ± 1.84	11.5 ± 1.32	32.3 ± 3.18	50.3 ± 2.73	17.3 ± 2.19	23.7 ± 1.20	44.0 ± 1.53	32.3 ± 2.40
	10	69.5 ± 2.90	27.5 ± 3.77	3.0 ± 1.08	50.0 ± 2.06	38.1 ± 2.54	11.9 ± 0.51	30.6 ± 1.84	38.3 ± 3.84	31.1 ± 2.48	34.0 ± 1.36***	21.4 ± 0.62	44.6 ± 1.10
	25	72.3 ± 2.85	25.0 ± 2.52	2.7 ± 0.67	52.7 ± 1.33	36.0 ± 1.15	11.3 ± 1.76	34.2 ± 2.87	47.8 ± 1.70	18.0 ± 3.14	25.5 ± 2.50*	55.2 ± 2.56	19.2 ± 4.50
1308	0.5	70.7 ± 2.03	25.0 ± 2.89	4.3 ± 0.88	53.0 ± 1.91	41.2 ± 2.78	5.8 ± 1.11	34.3 ± 0.33	44.0 ± 1.15	21.7 ± 1.20	31.2 ± 1.11***	32.3 ± 1.49	36.5 ± 0.87
	1	69.3 ± 1.20	29.7 ± 1.76	1.0 ± 0.58	51.0 ± 2.35	41.2 ± 1.80	7.8 ± 1.70	39.8 ± 2.46**	40.8 ± 1.44	19.5 ± 1.19	35.2 ± 2.35***	27.4 ± 2.32	37.4 ± 3.54
	5	71.0 ± 2.65	26.3 ± 3.18	2.7 ± 0.88	64.5 ± 2.33	30.0 ± 3.70	5.5 ± 1.55	59.2 ± 2.50***	20.5 ± 0.65	20.2 ± 2.06	45.8 ± 3.25***	14.8 ± 3.77	39.5 ± 1.50
	10	71.3 ± 4.10	26.7 ± 4.33	2.0 ± 0.58	53.3 ± 1.45	32.3 ± 0.67	14.3 ± 1.67	66.7 ± 1.76***	17.3 ± 2.33	16.0 ± 0.58	75.2 ± 2.25***	6.5 ± 1.85	18.2 ± 3.77
	25	75.3 ± 0.88	24.7 ± 0.88	0.0 ± 0.00	69.3 ± 1.45	29.3 ± 1.20	1.3 ± 0.88	69.0 ± 1.15***	28.0 ± 1.00	3.0 ± 0.58	75.7 ± 1.67***	20.3 ± 1.20	4.0 ± 0.58
1910	0.5	65.7 ± 2.85	31.0 ± 2.65	3.3 ± 0.67	44.7 ± 1.86	36.7 ± 1.20	18.7 ± 2.91	33.7 ± 1.20	57.0 ± 1.00	9.3 ± 2.19[s]	22.4 ± 3.13	35.1 ± 1.22	42.6 ± 2.15
	1	66.7 ± 3.53	29.0 ± 3.79	4.3 ± 0.33	45.4 ± 2.02	40.2 ± 2.53	14.2 ± 2.63	28.9 ± 2.26	44.6 ± 3.51	26.4 ± 5.39	26.8 ± 1.03**	39.5 ± 0.29	33.8 ± 0.75
	5	59.3 ± 2.03	38.0 ± 1.15	2.7 ± 0.88	61.8 ± 1.65	33.0 ± 2.12	5.2 ± 0.75	39.7 ± 4.67*	48.0 ± 2.65	12.3 ± 2.33	32.7 ± 1.59***	46.9 ± 3.12	20.5 ± 2.91
	10	68.0 ± 2.52	30.0 ± 2.08	2.0 ± 1.00	60.5 ± 2.06	37.8 ± 1.89	1.8 ± 0.48	59.0 ± 2.08***	37.3 ± 1.67	3.7 ± 0.67	59.0 ± 1.58***	34.0 ± 1.41	7.0 ± 1.22
	25	72.7 ± 2.50	24.3 ± 2.19	3.0 ± 1.53	70.5 ± 2.50**	27.2 ± 1.55	2.2 ± 1.60	56.3 ± 3.18***	40.0 ± 2.52	3.7 ± 0.67	69.8 ± 1.67***	25.8 ± 1.38	4.5 ± 1.19
1923	0.5	74.3 ± 1.67	24.3 ± 1.86	1.3 ± 0.33	53.5 ± 2.72	35.4 ± 2.53	11.0 ± 1.57	28.7 ± 0.33	53.0 ± 3.00	18.3 ± 2.67	18.3 ± 0.88	35.0 ± 1.53	46.7 ± 1.76
	1	74.3 ± 2.96	23.3 ± 2.03	2.3 ± 1.20	54.0 ± 2.08	37.7 ± 1.76	8.3 ± 0.33	27.6 ± 2.10	50.8 ± 3.00	21.6 ± 2.29	22.8 ± 1.80	34.0 ± 2.74	43.2 ± 1.31
	5	71.6 ± 2.32	25.4 ±1.82	3.0 ± 0.70	45.3 ± 0.67	46.7 ± 2.03	8.0 ± 1.53	27.0 ± 2.65	52.0 ± 3.06	21.0 ± 3.21	20.8 ± 2.02	38.0 ± 4.14	41.2 ± 4.48
	10	67.2 ± 1.70	29.3 ± 2.59	3.5 ± 1.04	46.5 ± 0.87	42.0 ± 1.78	11.5 ± 1.19	22.7 ± 1.20	43.3 ± 2.03	34.0 ± 1.53	26.0 ± 1.35**	45.5 ± 2.96	28.5 ± 2.50
	25	65.7 ± 1.45	31.0 ± 1.53	3.3 ± 0.33	48.3 ± 1.45	41.3 ± 2.19	10.3 ± 2.60	40.3 ± 0.67**	50.3 ± 2.19	9.3 ± 1.86	43.0 ± 3.49***	35.2 ± 0.95	21.8 ± 2.84

Supplementary Table S5 cont. In Vitro testing of *Clonostachys rosea* culture filtrate against *Meloidogyne incognita* egg development.

Treatment	Concentration	Day 1			Day 3			Day 6			Day 9		
		Developmental stage (%)			Developmental stage (%)			Developmental stage (%)			Developmental stage (%)		
	(media %, v/v)	Egg[1]	Pretzel[2]	J2[3]	Egg[4]	Pretzel[5]	J2[6]	Egg[7]	Pretzel[8]	J2[9]	Egg[10]	Pretzel[11]	J2[12]
**1952**	0.5	71.7 ± 1.20	26.7 ± 0.88	1.7 ± 0.33	54.3 ± 1.20	42.7 ± 1.76	3.0 ± 1.00	23.3 ± 1.33	42.0 ± 1.53	34.7 ± 1.20	19.0 ± 1.53	26.3 ± 2.33	54.7 ± 2.33
	1	68.5 ± 1.19	30.0 ± 1.83	1.5 ± 0.87	50.8 ± 1.25	41.8 ± 1.11	7.5 ± 1.04	33.7 ± 1.86	58.0 ± 1.53	8.3 ± 1.20	25.0 ± 2.00	40.7 ± 1.33	34.3 ± 0.67
	5	68.3 ± 0.33	27.0 ± 1.53	4.7 ± 1.67	57.8 ± 2.29	38.5 ± 2.60	3.8 ± 0.85	35.0 ± 5.51	42.0 ± 2.00	23.0 ± 7.23	21.7 ± 2.03	44.0 ± 1.53	34.3 ± 1.45
	10	68.3 ± 2.60	26.3 ± 2.73	5.3 ± 0.67	62.8 ± 2.02	33.5 ± 2.10	3.8 ± 0.48	56.0 ± 0.58***	42.0 ± 0.58	2.0 ± 0.00	59.0 ± 0.58***	38.3 ± 0.33	2.7 ± 0.67
	25	69.9 ± 1.07	29.8 ± 0.79	0.3 ± 0.33	78.5 ± 1.66***	19.2 ± 1.49	2.2 ± 0.63	60.3 ± 2.19***	38.0 ± 2.52	1.7 ± 0.33	68.7 ± 0.33***	29.0 ± 0.00	2.3 ± 0.33
**2084**	0.5	63.7 ± 2.03	31.6 ± 1.29	4.8 ± 1.29	50.2 ± 2.46	38.5 ± 3.59	11.2 ± 2.17	25.0 ± 306	54.7 ± 2.91	20.3 ± 0.67	18.3 ± 0.88	50.3 ± 1.45	31.3 ± 0.67
	1	58.0 ± 2.08	38.3 ± 1.86	3.7 ± 0.33	44.8 ± 1.38	46.0 ± 1.87	9.2 ± 1.80	29.3 ± 0.88	41.7 ± 4.18	29.0 ± 3.61	23.3 ± 0.67	37.3 ± 0.67	39.3 ± 0.67
	5	62.3 ± 1.67	34.7 ± 1.20	3.0 ± 0.58	56.3 ± 0.33	36.7 ± 1.20	7.0 ± 1.53	30.7 ± 2.73	38.7 ± 0.88	30.7 ± 3.33	22.0 ± 1.00	41.0 ± 2.00	37.0 ± 1.00
	10	62.0 ± 2.94	31.8 ± 2.36	6.2 ± 0.75	52.3 ± 1.20	38.3 ± 1.20	9.3 ± 0.67	37.2 ± 2.53	42.9 ± 7.39	19.9 ± 8.84	29.0 ± 1.53***	43.0 ± 0.58	28.0 ± 1.73
	25	61.7 ± 1.45	32.0 ±1.15	6.3 ± 0.33	61.0 ± 2.65	34.8 ± 1.89	4.2 ± 1.31	28.6 ± 1.83	38.9 ± 7.56	32.5 ± 7.58	24.0 ± 0.58	42.7 ± 1.20	33.3 ± 1.20
**2135**	0.5	67.5 ± 2.78	29.8 ± 2.69	2.8 ± 0.75	55.7 ± 2.03	36.3 ± 2.40	8.0 ± 0.58	31.0 ± 1.53	39.3 ± 1.20	29.7 ± 1.45	30.7 ± 0.33***	38.7 ± 1.76	30.7 ± 1.86
	1	61.3 ± 1.20	34.7 ± 1.76	4.0 ± 0.58	52.7 ± 0.67	35.7 ± 2.67	11.7 ± 3.33	28.7 ± 1.76	47.7 ± 2.73	23.7 ± 3.28	20.3 ± 0.63	43.2 ± 0.74	36.6 ± 0.99
	5	69.7 ± 1.76	28.3 ± 1.67	2.0 ± 0.58	59.0 ± 1.29	33.0 ± 2.27	8.0 ± 1.22	37.0 ± 1.53	48.3 ± 0.67	14.7 ± 1.86	31.3 ± 0.33***	40.7 ± 0.33	28.0 ± 0.58
	10	67.0 ± 2.00	30.0 ± 2.52	3.0 ± 0.58	44.0 ± 1.73	47.3 ± 0.88	8.7 ± 0.88	38.7 ± 1.76*	47.7 ± 2.60	13.7 ± 2.73	27.0 ± 0.58**	38.3 ± 0.33	34.7 ± 0.33
	25	75.0 ± 1.00	23.3 ± 0.88	1.7 ± 0.33	48.8 ± 2.46	46.8 ± 2.72	4.5 ± 0.65	24.3 ± 0.74	55.8 ± 5.29	20.0 ± 4.55	26.0 ± 1.53*	46.0 ± 2.00	28.0 ± 1.53
**2676**	0.5	71.0 ± 1.00	26.3 ± 1.20	2.7 ± 0.33	52.5 ± 1.50	36.5 ± 1.32	11.0 ± 0.41	28.0 ± 1.15	41.3 ± 3.38	30.7 ± 2.33	17.7 ± 1.20	46.0 ± 2.52	36.3 ± 1.33
	1	64.0 ± 1.15	31.7 ± 1.20	4.3 ± 0.88	51.3 ± 0.88	40.0 ± 1.00	8.7 ± 1.45	22.0 ± 1.15	52.7 ± 2.67	25.3 ± 1.76	22.0 ± 1.00	48.0 ± 0.58	30.0 ± 1.15
	5	63.8 ± 1.75	33.0 ± 1.58	3.2 ± 0.48	52.7 ± 0.88	35.7 ± 0.67	11.7 ± 1.20	21.3 ± 2.19	44.3 ± 1.45	34.3 ± 0.88	25.0 ± 0.58	50.3 ± 1.45	24.7 ± 2.03
	10	67.3 ± 0.67	31.0 ± 0.58	1.7 ± 0.88	53.8 ± 2.06	34.8 ± 3.79	11.5 ± 1.89	31.7 ± 1.20	47.7 ± 1.45	20.7 ± 2.19	32.3 ± 1.45***	48.7 ± 0.88	19.0 ± 1.00
	25	62.0 ± 2.00	33.3 ± 2.67[c]	4.7 ± 0.67	47.8 ± 0.63	40.5 ± 1.44	11.8 ± 1.18	34.7 ± 1.76	38.3 ± 2.91	27.0 ± 1.15	28.3 ± 1.20**	37.7 ± 1.45	34.0 ± 2.65

Values represent mean percentage ± SE of eggs, pretzels, and second-stage juveniles (J2) across biological replicates. Percentages were calculated separately for each replicate from the observed numbers of eggs, pretzels, and J2. The number of individuals counted therefore varied among replicates. The nematode control consisted of eggs maintained without fungal culture filtrate. Statistical comparisons for egg development were performed using a binomial generalized linear model with Egg versus non-Egg (Pretzel + J2) as the response,

with Day, Treatment, and their interaction as fixed effects. Pairwise comparisons between each treatment and the nematode control were performed within each day, with Holm adjustment. Asterisks indicate significant differences from the nematode control within the corresponding day (* $P \leq 0.05$, ** $P \leq 0.01$, *** $P \leq 0.001$).

Supplementary Table S6. In Vitro testing of *Clonostachys rosea* culture filtrate against *Meloidogyne incognita* J2.

Treatment	Concentration	Day 1			Day 2			Day 3			Day 7		
		J2 Motility (%)			J2 Motility (%)			J2 Motility (%)			J2 Motility (%)		
	(media %, v/v)	Active	Inhibited	Immobile	Active	Inhibited	Immobile	Active	Inhibited	Immobile	Active	Inhibited	Immobile
**Control**	0	100.0 ± 0.00	0.0 ± 0.00	0.0 ± 0.00	97.4 ± 0.4	1.6 ± 0.2	1.0 ± 0.3	94.0 ± 0.4	3.6 ± 0.5	2.4 ± 0.7	86.2 ± 0.9	7.0 ± 0.3	6.8 ± 0.8
**PHP1710**	0.5	99.0 ± 0.4	0.5 ± 0.3	0.5 ± 0.3	94.5 ± 1.0	4.0 ± 1.2	1.5 ± 0.3	83.8 ± 1.1***	12.8 ± 0.6	3.5 ± 0.5	45.5 ± 0.9***	40.3 ± 0.5	14.2 ± 0.8
	1	99.8 ± 0.2	0.2 ± 0.2	0.0 ± 0.0	89.8 ± 1.0***	7.8 ± 0.6	2.5 ± 0.5	83.8 ± 1.8***	14.0 ± 1.4	2.2 ± 0..6	51.2 ± 1.6***	37.8 ± 2.0	11.0 ± 0.6
	5	94.5 ± 1.0	4.5 ± 0.3	1.0 ± 0.7	84.2 ± 0.9***	8.6 ± 0.5	7.1 ± 1.1	75.2 ± 1.5***	21.5 ± 0.5	3.2 ± 1.2	38.2 ± 1.2***	44.3 ± 2.5	17.5 ± 1.2
	10	92.2 ± 0.9*	5.8 ± 0.8	2.0 ± 0.4	82.8 ± 1.4***	11.2 ± 1.2	6.0 ± 0.7	76.0 ± 0.4***	19.2 ± 0.9	4.8 ± 0.8	34.8 ± 1.0***	56.0 ± 2.0	9.2 ± 1.2
	25	88.5 ± 1.3***	8.8 ± 0.6	2.8 ± 0.8	82.5 ± 2.7***	13.0 ± 1.8	4.5 ± 1.2	76.3 ± 2.3***	16.5 ± 1.5	7.2 ± 1.1	30.0 ± 1.2***	60.8 ± 1.5	9.2 ± 0.5
**1308**	0.5	97.8 ± 0.8	1.5 ± 0.6	0.8 ± 0.5	91.0 ± 1.2***	6.5 ± 0.9	2.5 ± 0.5	91.0 ± 0.4	7.3 ± 0.2	1.8 ± 0.2	47.2 ± 1.1***	42.8 ± 1.2	10.0 ± 1.0
	1	96.5 ± 0.6	3.3 ± 0.6	0.2 ± 0.2	89.0 ± 0.6***	6.5 ± 0.3	4.5 ± 0.3	86.8 ± 0.6**	12.0 ± 0.4	1.2 ± 0.2	41.0 ± 0.4***	51.8 ± 1.8	7.2 ± 1.4
	5	85.5 ± 1.4***	12.5 ± 1.4	2.0 ± 0.0	0.0 ± 0.0***	14.0 ± 0.8	86.0 ± 0.8	2.5 ± 0.9***	44.0 ± 13.0	53.5 ± 13.9	31.0 ± 1.7***	64.2 ± 1.4	4.8 ± 1.7
	10	0.0 ± 0.0***	23.0 ± 2.3	77.0 ± 2.3	0.0 ± 0.0***	12.2 ± 1.0	87.8 ± 1.0	0.0 ± 0.0***	13.2 ± 0.6	86.8 ± 0.6	56.5 ± 0.9***	37.3 ± 1.0	6.2 ± 0.2
	25	0.0 ± 0.0***	13.5 ± 1.6	86.5 ± 1.6	0.0 ± 0.0***	13.0 ± 0.4	87.0 ± 0.4	0.0 ± 0.0***	7.2 ± 0.6	92.8 ± 0.6	0.0 ± 0.0***	4.8 ± 0.2	95.2 ± 0.2
**1910**	0.5	88.0 ± 0.6***	10.5 ± 0.9	1.5 ± 0.6	95.8 ± 0.6	2.8 ± 0.5	1.5 ± 0.5	93.0 ± 0.4	6.0 ± 0.4	1.0 ± 0.0	48.0 ± 1.2***	48.8 ± 1.5	3.2 ± 0.5
	1	81.5 ± 1.8***	14.5 ± 1.8	4.0 ± 0.9	93.2 ± 0.2*	5.0 ± 0.0	1.8 ± 0.2	90.0 ± 0.0	9.2 ± 0.2	0.8 ± 0.2	32.8 ± 1.5***	63.2 ± 1.8	4.0 ± 0.6
	5	0.0 ± 0.0***	13.2 ± 1.1	86.8 ± 1.1	0.0 ± 0.0***	17.0 ± 2.1	83.0 ± 2.1	0.0 ± 0.0***	10.8 ± 0.8	89.2 ± 0.8	42.5 ± 0.9***	55.5 ± 1.4	2.0 ± 0.6
	10	0.0 ± 0.0***	21.2 ± 0.6	78.8 ± 0.6	0.0 ± 0.0***	15.8 ± 0.6	84.2 ± 0.6	0.0 ± 0.0***	10.5 ± 0.9	89.5 ± 0.9	0.0 ± 0.0***	11.2 ± 0.6	88.8 ± 0.6
	25	0.0 ± 0.0***	28.8 ± 4.60	71.2 ± 4.6	0.0 ± 0.0***	18.0 ± 1.0	82.0 ± 1.0	0.0 ± 0.0***	9.0 ± 0.7	91.0 ± 0.7	0.0 ± 0.0***	8.2 ± 0.6	91.8 ± 0.6
**1923**	0.5	98.0 ± 0.4	1.8 ± 0.6	0.2 ± 0.2	95.0 ± 0.0	4.0 ± 0.0	1.0 ± 0.0	90.5 ± 0.9	9.0 ± 1.0	0.5 ± 0.3	44.2 ± 1.1***	52.0 ± 0.4	3.8 ± 0.8
	1	95.0 ± 0.4	4.2 ± 0.2	0.8 ± 0.2	96.0 ± 0.4	3.5 ± 0.3	0.5 ± 0.5	89.8 ± 0.6	10.0 ± 0.4	0.2 ± 0.2	33.0 ± 0.4***	62.0 ± 0.0	5.0 ± 0.4
	5	72.0 ± 0.4***	20.5 ± 1.0	7.5 ± 0.9	14.0 ± 8.2***	35.5 ± 13.7	50.5 ± 6.0	36.2 ± 8.1***	45.2 ± 3.4	18.5 ± 4.7	41.8 ± 0.6***	54.0 ± 1.4	4.2 ± 0.8
	10	42.5 ± 3.40***	35.0 ± 2.4	22.5 ± 4.2	0.0 ± 0.0***	21.5 ± 1.3	78.5 ± 1.3	0.0 ± 0.0***	13.5 ± 1.3	86.5 ± 1.3	52.5 ± 0.9***	39.0 ± 1.2	8.5 ± 1.5
	25	0.0 ± 0.0***	14.0 ± 2.1	86.0 ± 2.1	0.0 ± 0.0***	12.5 ± 0.9	87.5 ± 0.9	0.0 ± 0.0***	11.5 ± 0.9	88.5 ± 0.9	0.0 ± 0.0***	8.2 ± 0.5	91.8 ± 0.5

Supplementary Table S6 cont. In Vitro testing of *Clonostachys rosea* culture filtrate against *Meloidogyne incognita* J2.

Treatment	Concentration	Day 1			Day 2			Day 3			Day 7		
		J2 Motility (%)			J2 Motility (%)			J2 Motility (%)			J2 Motility (%)		
	(media %, v/v)	Active	Inhibited	Immobile	Active	Inhibited	Immobile	Active	Inhibited	Immobile	Active	Inhibited	Immobile
1952	0.5	89.0 ± 2.1***	10.0 ± 2.2	1.0 ± 0.4	92.2 ± 0.8**	6.0 ± 0.4	1.8 ± 0.5	91.5 ± 0.5	8.0 ± 0.4	0.5 ± 0.3	40.8 ± 1.1***	55.4 ± 1.2	3.8 ± 0.5
	1	77.5 ± 0.9***	18.8 ± 1.8	3.8 ± 0.9	83.5 ± 1.5***	13.0 ± 1.2	3.5 ± 0.9	83.0 ± 1.0***	13.3 ± 0.8	3.8 ± 0.2	33.2 ± 0.2***	58.3 ± 0.3	8.5 ± 0.3
	5	0.0 ± 0.0***	18.5 ± 1.7	81.5 ± 1.7	0.0 ± 0.0***	17.7 ± 0.8	82.3 ± 0.8	0.0 ± 0.0***	19.8 ± 2.1	80.2 ± 2.1	42.5 ± 0.5***	52.5 ± 0.5	5.0 ± 0.8
	10	0.0 ± 0.0***	19.5 ± 0.9	80.5 ± 0.9	0.0 ± 0.0***	19.0 ± 1.0	81.0 ± 1.0	0.0 ± 0.0***	9.8 ± 0.6	90.2 ± 0.6	57.2 ± 0.6***	33.6 ± 1.3	9.2 ± 0.7
	25	0.0 ± 0.0***	20.0 ± 1.7	80.0 ± 1.7	0.0 ± 0.0***	18.8 ± 1.9	81.2 ± 1.9	0.0 ± 0.0***	11.8 ± 1.1	88.2 ± 1.1	0.0 ± 0.0***	12.5 ± 0.9	87.5 ± 0.9
2084	0.5	98.5 ± 0.3	1.5 ± 0.3	0.0 ± 0.0	94.2 ± 0.6	4.8 ± 0.6	1.0 ± 0.0	87.5 ± 2.1**	9.5 ± 2.2	3.0 ± 0.6	50.2 ± 0.6***	43.5 ± 0.3	6.3 ± 0.8
	1	98.2 ± 0.2	1.3 ± 0.2	0.5 ± 0.3j	96.0 ± 0.0	2.5 ± 0.5	1.5 ± 0.5	79.5 ± 0.9***	18.5 ± 0.5	2.0 ± 0.4	38.0 ± 0.6***	52.2 ± 1.4	9.8 ± 1.1
	5	97.8 ± 0.2	1.8 ± 0.2	0.5 ± 0.3	84.8 ± 1.0***	13.2 ± 0.6	2.0 ± 0.4	68.6 ± 1.2***	27.7 ± 1.4	3.7. ± 0.8	45.2 ± 1.6***	44.0 ± 0.7	10.8 ± 1.2
	10	88.2 ± 0.2***	11.2 ± 0.5	0.5 ± 0.3	89.5 ± 0.9***	7.8 ± 0.7	2.8 ± 0.2	56.0 ± 3.0***	40.2 ± 2.1	3.8 ± 0.9	48.8 ± 0.6***	41.2 ± 0.2	10.0 ± 0.0
	25	12.0 ± 3.2***	50.4 ± 6.4	37.6 ± 9.4	0.0 ± 0.0***	15.8 ± 1.1	84.2 ± 1.1	0.0 ± 0.0***	13.0 ± 1.7	87.0 ± 1.7	47.2 ± 2.5***	47.8 ± 2.1	5.0 ± 0.4
2135	0.5	89.2 ± 0.6***	8.2 ± 0.2	2.5 ± 0.5	86.3 ± 1.1***	9.2 ± 1.0	4.5 ± 0.3	77.5 ± 1.8***	20.2 ± 2.2	2.2 ± 0.9	24.4 ± 0.8***	37.4 ± 0.2	38.2 ± 0.3
	1	90.5 ± 0.5***	8.2 ± 0.5	1.2 ± 0.2	81.2 ± 0.6***	14.2 ± 1.0	4.5 ± 1.0	61.8 ± 0.6***	33.2 ± 0.2	5.0 ± 0.8	33.2 ± 1.2***	49.0 ± 1.2	17.8 ± 0.8
	5	87.0 ± 0.0***	11.0 ± 0.0	2.0 ± 0.0	74.5 ± 0.3***	19.2 ± 0.8	6.2 ± 0.5	50.0 ± 0.8***	40.8 ± 1.4	9.2 ± 1.0	36.8 ± 1.4***	42.0 ± 1.0	21.2 ± 2.3
	10	78.5 ± 0.9***	15.8 ± 1.1	5.8 ± 0.2	57.5 ± 1.3***	24.8 ± 2.0	17.8 ± 2.0	30.5 ± 1.7***	61.8 ± 1.3	7.8 ± 1.2	42.6 ± 0.4***	34.4 ± 0.3	23.0 ± 1.4
	25	30.5 ± 11.7***	33.5 ± 3.0	36.0 ± 11.3	73.5 ± 1.3***	20.8 ± 1.9	5.8 ± 1.0	44.0 ± 1.4***	45.8 ± 0.5	10.2 ± 1.4	30.5 ± 1.3***	43.0 ± 1.2	26.5 ± 1.7
2676	0.5	94.5 ± 1.3	4.5 ± 1.2	1.0 ± 0.4	88.5 ± 0.5***	10.2 ± 0.2	1.2 ± 0.2	90.5 ± 0.9	8.2 ± 1.1	1.2 ± 0.2	36.8 ± 0.6***	44.2 ± 1.6	19.0 ± 2.3
	1	90.0 ± 0.4***	8.5 ± 0.5	1.5 ± 0.3	86.0 ± 0.4***	12.5 ± 0.9	1.5 ± 0.5	85.2 ± 0.6***	12.8 ± 0.6	2.0 ± 0.0	36.0 ± 0.4***	39.8 ± 1.2	24.2 ± 1.4
	5	86.0 ± 1.4***	12.0 ± 1.2	2.0 ± 0.6	78.8 ± 0.6***	16.8 ± 0.2	4.5 ± 0.5	78.2 ± 1.0***	18.0 ± 0.6	3.8 ± 1.1	41.0 ± 0.6***	37.2 ± 1.7	21.8 ± 2.1
	10	75.5 ± 0.3***	22.0 ± 0.7	2.5 ± 0.5	77.5 ± 2.2***	15.8 ± 1.7	6.8 ± 0.6	56.5 ± 1.2***	35.7 ± 0.6	7.8 ± 1.3	42.5 ± 1.9***	36.5 ± 0.3	21.0 ± 1.4
	25	60.8 ± 1.1***	36.2 ± 1.4	3.0 ± 0.7	75.5 ± 0.9***	19.8 ± 1.0	4.8 ± 0.5	54.8 ± 1.4***	39.2 ± 2.8	6.0 ± 1.4	35.0 ± 0.4***	43.5 ± 1.8	21.5 ± 1.4

Values represent mean percentage ± SE of active, inhibited, and immobile second-stage juveniles (J2) across biological replicates. Percentages were calculated separately for each replicate from the observed numbers of active, inhibited, and immobile J2; therefore, the total number of individuals counted varied among replicates. The nematode control consisted of J2 maintained without fungal culture filtrate. Statistical comparisons of J2 activity were performed using bias-reduced binomial logistic regression, with the numbers of active and non-active J2 (inhibited + immobile) as the response and Treatment as the explanatory variable, fitted separately for each observation day. Pairwise comparisons between each filtrate

treatment and the nematode control were performed within each day, with Holm adjustment for multiple comparisons. Asterisks indicate significant differences from the nematode control within the corresponding day (* $P \leq 0.05$, ** $P \leq 0.01$, *** $P \leq 0.001$).

Supplementary Table S7. Gall index (GI) and plant biomass of cucumber plants 21 days after inoculation with *Meloidogyne incognita* J2 pre-exposed for seven days to culture filtrates of *Clonostachys rosea* isolates at different concentrations.

**Treatment (media %, v/v)**		**[1]GI (mean ± SE)**	**Dunn-Holm vs. Nematode control**	**[2]Shoot fresh weight (g)**	**[3]Root fresh weight (g)**
**Nematode Control**	**0**	7.00 ± 0.00	-	1.04 ± 0.03^{a}	1.00 ± 0.03^{ab}
**PHP1710**	**0.5**	6.80 ± 0.20	ns	0.88 ± 0.06^{a}	0.80 ± 0.07^{a}
	**1.0**	6.60 ± 0.25	ns	1.07 ± 0.03^{b}	1.04 ± 0.05^{a}
	**5.0**	7.00 ± 0.00	ns	1.07 ± 0.04^{b}	0.92 ± 0.06^{a}
	**10**	6.40 ± 0.25	ns	1.16 ± 0.06^{b}	1.02 ± 0.03^{a}
	**25**	5.60 ± 0.25	0.0308*	1.06 ± 0.08^{b}	0.92 ± 0.05^{a}
**1308**	**0.5**	6.40 ± 0.25	ns	0.88 ± 0.06^{a}	0.96 ± 0.02^{ab}
	**1.0**	7.00 ± 0.00	ns	0.89 ± 0.06^{a}	0.97 ± 0.03^{ab}
	**5.0**	7.00 ± 0.00	ns	0.96 ± 0.04^{a}	0.88 ± 0.02^{ab}
	**10**	6.60 ± 0.25	ns	0.98 ± 0.07^{a}	0.95 ± 0.03^{ab}
	**25**	4.40 ± 0.25	0.0009***	0.92 ± 0.06^{a}	0.76 ± 0.01^{ab}
**1910**	**0.5**	6.60 ± 0.25	ns	0.92 ± 0.06^{ab}	1.03 ± 0.04^{a}
	**1.0**	6.60 ± 0.25	ns	0.82 ± 0.03^{a}	1.03 ± 0.07^{a}
	**5.0**	6.40 ± 0.25	ns	0.94 ± 0.02^{ab}	1.02 ± 0.04^{a}
	**10**	5.60 ± 0.25^{c}	0.0308*	0.88 ± 0.03^{ab}	0.83 ± 0.01^{a}
	**25**	4.60 ± 0.25	0.0011**	1.02 ± 0.07^{b}	0.96 ± 0.05^{a}
**1923**	**0.5**	6.60 ± 0.25	ns	1.06 ± 0.05^{a}	0.82 ± 0.10^{ab}
	**1.0**	6.60 ± 0.25	ns	1.08 ± 0.04^{a}	0.85 ± 0.04^{ab}
	**5.0**	6.60 ± 0.25	ns	1.15 ± 0.04^{a}	0.98 ± 0.04^{ab}
	**10**	6.60 ± 0.25	ns	1.11 ± 0.06^{a}	0.95 ± 0.04^{ab}
	**25**	6.60 ± 0.25	ns	1.09 ± 0.02^{a}	0.91 ± 0.02^{ab}
**1952**	**0.5**	6.60 ± 0.25	ns	1.09 ± 0.03^{a}	0.93 ± 0.04^{b}
	**1.0**	6.40 ± 0.25	ns	1.11 ± 0.06^{a}	0.80 ± 0.02^{b}
	**5.0**	6.40 ± 0.25	ns	1.08 ± 0.02^{b}	0.82 ± 0.04^{b}
	**10**	6.80 ± 0.20	ns	0.90 ± 0.08^{ab}	0.77 ± 0.02^{b}
	**25**	6.40 ± 0.25	ns	0.98 ± 0.04^{ab}	0.82 ± 0.02^{b}
**2084**	**0.5**	7.00 ± 0.00	ns	1.05 ± 0.03^{a}	0.77 ± 0.08^{ab}
	**1.0**	7.00 ± 0.00	ns	0.99 ± 0.02^{a}	0.81 ± 0.08^{ab}
	**5.0**	7.00 ± 0.00	ns	0.98 ± 0.04^{a}	0.85 ± 0.05^{ab}
	**10**	6.40 ± 0.25	ns	0.93 ± 0.01^{a}	0.92 ± 0.04^{ab}
	**25**	5.60 ± 0.25	0.0308*	1.01 ± 0.03^{a}	0.96±0.04^{ab}
**2135**	**0.5**	6.60 ± 0.25	ns	1.08 ± 0.06^{a}	0.98 ± 0.04^{a}
	**1.0**	6.40 ± 0.25	ns	0.99 ± 0.03^{a}	0.98 ± 0.01^{a}
	**5.0**	6.60 ± 0.25	ns	1.04 ± 0.03^{a}	1.00 ± 0.06^{a}
	**10**	6.20 ± 0.25	ns	1.14 ± 0.07^{a}	0.91 ± 0.06^{a}
	**25**	5.60 ± 0.25	0.0308*	0.99 ± 0.01^{a}	0.93 ± 0.04^{a}
**2676**	**0.5**	6.40 ± 0.25	ns	0.98 ± 0.03^{a}	0.95 ± 0.03^{a}
	**1.0**	6.40 ± 0.25	ns	1.06 ± 0.02^{a}	0.97 ± 0.06^{a}
	**5.0**	6.00 ± 0.00	ns	1.00 ± 0.02^{a}	1.07 ± 0.07^{a}
	**10**	6.60 ± 0.25	ns	1.02 ± 0.05^{a}	1.00 ± 0.02^{a}
	**25**	6.40 ± 0.25	ns	0.99 ± 0.01^{a}	0.96 ± 0.04^{a}

[1]GI: Values are means ± SE (n = 5). P-values represent Dunn's post-hoc comparisons with the nematode control, adjusted using the Holm method. ns, not significant; *$P < 0.05$, **$P < 0.01$, ***$P < 0.001$.
[2,3]Shoot and Root fresh weights: Values are means ± SE ($n = 5$). Different lowercase letters within the same treatment indicate significant differences among concentrations according to Tukey's multiple-comparison test ($P < 0.05$).

Supplementary Table S8. Small scale trial: gall index (GI), root biomass, and *Meloidogyne incognita* reproductiom parameters following treatment with selected *Clonostachys rosea* isolates.

Treatment	[1]GI	[2]Root Weight (g)	[3]Egg mass	[4]Egg Count	[5]Egg/Egg mass
Nematode control	4.75 ± 0.48	6.32 ± 0.30	544 ± 88	317,708 ± 41,717	594 ± 38
1308	5.25 ± 0.37	6.05 ± 0.30	602 ± 34	365,292 ± 17,937	610 ± 34
1910	5.63 ± 0.26	6.00 ± 0.30	533 ± 72	330,708 ± 17,940	6456 ± 61
1952	5.00 ± 0.29	6.07 ± 0.30	461 ± 33	293,375 ± 28,035	653 ± 87
2084	5.75 ± 0.29	7.18 ± 0.30	507 ± 65	377,292 ± 38,143	757 ± 61

[1]GI: Values represent means ± SE. Gall ratings did not differ significantly among treatments (Kruskal–Wallis test, $\chi^2 = 6.16$, df = 4, $P = 0.188$).

[2]Root fresh weight differed significantly among treatments (one-way ANOVA, $F_{4,35} = 2.76$, $P = 0.043$), but no pairwise differences were significant after Tukey adjustment ($P \geq 0.264$).

[3]Egg mass did not differ significantly among treatments (one-way ANOVA, $F_{4,15} = 0.69$, $P = 0.608$).

[4]Egg count did not differ significantly among treatments (one-way ANOVA, $F_{4,15} = 1.28$, $P = 0.321$).

[5]Egg/Egg mass did not differ significantly among treatments (one-way ANOVA, $F_{4,15} = 1.15$, $P = 0.372$)

Supplementary Table S9. Root gall index (GI) of tomato plants (*Solanum lycopersicum* cv. Roterno F1 RZ grafted onto Maxiforf) following treatment with *Clonostachys rosea* and inoculation with *Meloidogyne incognita* in a large-scale trial.

Treatment	GI			
	**Week 6**	**Week 10**	**Week 14**	**Week 18**
C	2.50 ± 0.29[a]	3.75 ± 0.25[a]	4.25 ± 0.25[a]	5.75 ± 0.25[a]
1308	2.25 ± 0.25[a]	3.25 ± 0.25[a]	4.25 ± 0.25[a]	5.50 ± 0.29[a]
1910	1.50 ± 0.29[a]	2.75 ± 0.25[a]	3.50 ± 0.29[a]	5.00 ± 0.41[a]
1952	1.75 ± 0.25[a]	2.75 ± 0.25[a]	4.25 ± 0.25[a]	5.33 ± 0.67[a]
2084	1.00 ± 0.00[b]	2.50 ± 0.29[b]	3.75 ± 0.25[a]	4.50 ± 0.29[a]

Values are means ± SE. Different lowercase letters within a column indicate significant differences from the nematode control based on Dunn's test with Holm adjustment ($P < 0.05$).

Supplementary Table S10. Average branch length of tomato plants (*Solanum lycopersicum* cv. Roterno F1 RZ grafted onto Maxifort) following treatment with *Clonostachys rosea* and inoculation with *Meloidogyne incognita* in a large-scale trial.

**Treatment**	**Average branch Length (cm)**			
	**Week 6**	**Week 10**	**Week 14**	**Week 18**
C	169.62 ± 3.88	301.12 ± 2.95	362.88 ± 11.18	478.50 ± 13.29
1308	158.38 ± 9.88	288.62 ± 7.32	367.75 ± 3.74	487.75 ± 9.06
1910	159.50 ± 3.97	282.88 ± 8.46	370.50 ± 3.23	489.62 ± 3.21
1952	161.75 ± 3.20	307.25 ± 2.29	359.00 ± 3.52	473.75 ± 19.99
2084	152.50 ± 3.21	296.88 ± 8.60	371.25 ± 9.80	464.62 ± 24.10

Values are means ± SE (n = 4). Average branch length was calculated as the mean of the left and right branch lengths.

Supplementary Table S11. Total number of second-stage juveniles recovered from soil in early (week 6), mid (week 10), and late (week 18) stages of tomato growth (*Solanum lycopersicum* cv. Roterno F1 RZ grafted onto Maxifort) following treatment with *Clonostachys rosea* and inoculation with *Meloidogyne incognita* in a large-scale trial.

**Treatment**	**Juveniles in 100 cm^3 of soil**		
	**Week 10**	**Week 14**	**Week 18**
C	53.1 ± 26.8	197.7 ± 20.3	2772.0 ± 338.2
1308	63.1 ± 19.7	237.2 ± 24.1	2097.3 ± 372.3
1910	48.6 ± 14.0	212.7 ± 14.7	2362.7 ± 590.7
1952	52.9 ± 17.8	206.7 ± 30.4	2602.7 ± 142.0
2084	66.9 ± 15.7	245.3 ± 41.3	1404.0 ± 225.9

Values represent mean ± SE (n = 4). No significant differences among treatments were detected at any sampling date (Kruskal–Wallis test, $P > 0.05$).

Supplementary Table S12. Cumulative fruit production and fruit weight of tomato plants (*Solanum lycopersicum* cv. Roterno F1 RZ grafted onto Maxifort) following treatment with *Clonostachys rosea* selected isolates and inoculation with *Meloidogyne incognita* in a large-scale trial.

Treatment	Cumulative fruit number (fruits plant^{-1})	Cumulative fruit weight (kg plant^{-1})	Mean fruit weight (g fruit^{-1})	Difference vs. Nematode control
Nematode control	123.0 ± 1.5	7.32 ± 0.29	59.5 ± 1.8	-
1308	132.2 ± 8.9	9.02 ± 0.65	68.2 ± 0.4	+23.2%
1910	133.5 ± 5.4	8.70 ± 0.64	65.0 ± 3.1	+18.9%
1952	122.5 ± 5.5	7.87 ± 0.50	64.1 ± 2.0	+7.5%
2084	133.5 ± 3.3	8.84 ± 0.28	66.2 ± 1.6	+20.8%

Values are means ± SE. Cumulative fruit number did not differ significantly among treatments (ANOVA, $F_{4,15}$ = 1.07, P = 0.406), nor did cumulative fruit weight ($F_{4,15}$ = 2.10, P = 0.131) or mean fruit weight ($F_{4,15}$ = 2.73, P = 0.069). Yield increase was calculated relative to the nematode control as [(treatment mean − control mean) / control mean] × 100 and represents a numerical difference, not a statistically significant treatment effect.

Supplementary Table S13. Fruit production and individual fruit weight at each harvest of tomato plants (*Solanum lycopersicum* cv. Roterno F1 RZ grafted onto Maxifort) treated with *Clonostachys rosea* selected isolates in a large-scale trial.

Harvest date	Treatment	Fruit number (fruits plant^{-1})	Individual fruit weight (g fruit^{-1})	vs. Nematode control
20.06.2024	Nematode control	15.5 ± 3.1	69.1 ± 3.4	-
	1308	19.2 ± 2.4	68.1 ± 4.7	ns
	1910	23.0 ± 2.5	73.1 ± 2.9	ns
	1952	16.8 ± 2.0	**80.6 ± 2.3***	ns
	2084	19.5 ± 2.6	78.6 ± 4.0	***P* = 0.0487**
27.06.2024	Nematode control	24.5 ± 1.5	76.1 ± 1.4	-
	1308	23.8 ± 1.9	80.3 ± 0.6	ns
	1910	23.8 ± 4.9	81.8 ± 4.1	ns
	1952	21.0 ± 2.7	76.4 ± 3.9	ns
	2084	24.8 ± 0.9	81.5 ± 4.0	ns
04.07.2024	Nematode control	18.0 ± 1.6	67.7 ± 2.7	-
	1308	19.5 ± 1.6	72.2 ± 2.7	ns
	1910	17.8 ± 1.3	68.1 ± 2.7	ns
	1952	17.8 ± 1.8	66.3 ± 2.7	ns
	2084	17.8 ± 1.8	67.6 ± 2.7	ns
18.07.2024	Nematode control	18.0 ± 2.0	57.0 ± 2.8	-
	1308	19.5 ± 1.3	**71.0 ± 2.8***	***P* = 0.0100**
	1910	17.5 ± 2.0	66.4 ± 2.8	ns
	1952	18.5 ± 2.0	65.6 ± 2.8	ns
	2084	19.5 ± 1.3	**70.3 ± 2.8***	***P* = 0.0124**
25.07.2024	Nematode control	16.0 ± 1.6	53.9 ± 2.7	-
	1308	20.8 ± 2.1	**73.1 ± 2.7***	***P* = 0.0002**
	1910	18.3 ± 2.1	57.0 ± 2.7	ns
	1952	17.0 ± 1.4	60.1 ± 2.7	ns
	2084	19.0 ± 1.6	**65.5 ± 2.7***	***P* = 0.0351**
31.07.2024	Nematode control	15.5 ± 1.0	42.3 ± 2.7	-
	1308	18.5 ± 1.0	**55.8 ± 2.7***	***P* = 0.0145**
	1910	16.3 ± 1.3	47.5 ± 2.7	ns
	1952	17.3 ± 1.5	51.3 ± 2.7	ns
	2084	16.3 ± 1.5	48.6 ± 2.7	ns
06.08.2024	Nematode control	15.0 ± 1.6	35.2 ± 2.8	-
	1308	17.5 ± 1.8	45.8 ± 2.8	ns
	1910	17.0 ± 1.3	**47.2 ± 2.8***	***P* = 0.0364**
	1952	16.5 ± 1.5	40.1 ± 2.8	ns
	2084	16.5 ± 1.3	42.2 ± 2.8	ns

Values are means ± SE (n = 4 plants per treatment and harvest). Individual fruit weight was calculated for each plant as total fruit weight divided by the number of fruits harvested from that plant. The vs. control column shows Holm-adjusted *P*-values for treatment-vs-nematode-control comparisons within each harvest date. ns = not significant ($P \geq 0.05$). The individual fruit-weight analysis was performed using a linear mixed-effects model with treatment, harvest date and their interaction as fixed effects and plant as a random effect. The treatment × harvest-date interaction was significant (χ^2_{24} = 50.55, P = 0.0012). Fruit number was analysed using a negative binomial generalized linear model; treatment was not significant (χ^2_4 = 6.84, P = 0.145), while harvest date was significant (χ^2_6 = 21.71, P = 0.0014), with no treatment × harvest-date interaction (χ^2_{24} = 13.70, P = 0.953).

Supplementary Table S14. Development of *Meloidogyne incognita* eggs during incubation.

Day	Egg (%)	Pretzel (%)	J2 (%)
**0**	100.0 ± 0.00	0.00 ± 0.00	0.00 ± 0.00
**3**	74.17 ± 0.7	25.00 ± 0.52	0.83± 0.54
**5**	60.00 ± 1.6	38.83± 1.45	1.17 ± 0.31
**7**	33.33 ± 1.8	62.17± 1.87	4.50 ± 0.85
**10**	19.33 ± 1.56	55.50 ± 2.79	25.33 ± 3.20
**14**	13.33 ± 0.71	7.83 ± 0.91	78.83 ± 0.76

Values represent mean percentages ± SE of six replicates. Egg development was assessed by classifying individuals as eggs, pretzel-stage embryos, or emerged second-stage juveniles (J2).
